# A Comprehensive Review of Substitutional Silicon-Doped C_60_ Fullerenes and Their Endohedral/Exohedral Complexes: Synthetic Strategies and Molecular Modeling Approaches

**DOI:** 10.3390/molecules30193912

**Published:** 2025-09-28

**Authors:** Monika Zielińska-Pisklak, Patrycja Siekacz, Zuzanna Stokłosa, Łukasz Szeleszczuk

**Affiliations:** 1 Department of Pharmaceutical Chemistry and Biomaterials, Faculty of Pharmacy, Medical University of Warsaw, Banacha 1 Str., 02-093 Warsaw, Poland; mpisklak@wum.edu.pl; 2 Department of Organic and Physical Chemistry, Medical University of Warsaw, 1 Banacha Str., 02-097 Warsaw, Poland; s091854@student.wum.edu.pl (P.S.); s091860@student.wum.edu.pl (Z.S.)

**Keywords:** silicon-doped C_60_ fullerenes, heterofullerenes, substitutional doping, endohedral complexes, exohedral functionalization, molecular modeling, density functional theory (DFT), nanocarriers, drug delivery, silicon

## Abstract

Silicon-doped C_60_ fullerenes represent a distinctive class of heterofullerenes with tunable structural, electronic, and chemical properties arising from substitutional incorporation of Si atoms into the carbon cage. This review provides a comprehensive analysis of substitutional Si–C_60_ systems and their endohedral and exohedral complexes, with emphasis on synthesis strategies, structural features, and theoretical investigations. Experimental methods, including laser vaporization and arc discharge of Si-containing graphite targets, have enabled the preparation of Si-doped fullerenes, although challenges remain in controlling the dopant number, position, and distribution. Computational studies, dominated by density functional theory and molecular dynamics simulations, elucidate the effects of Si substitution on cage geometry, HOMO–LUMO modulation, charge localization, aromaticity, and finite-temperature stability. Exohedral functionalization and endohedral encapsulation of Si-doped cages significantly enhance their potential for applications in sensing, catalysis, energy storage, and nanomedicine. Si incorporation consistently strengthens adsorption of small molecules, pharmaceuticals, biomolecules, and environmental pollutants, often transforming weak physisorption into strong chemisorption with pronounced electronic and spectroscopic changes. The synergistic insights from experimental and theoretical work establish Si-doped fullerenes as versatile, electronically responsive nanoplatforms, offering a balance between stability, tunability, and reactivity, and highlighting future opportunities for targeted synthesis and application-specific design.

## 1. Introduction

**Figure 1:**
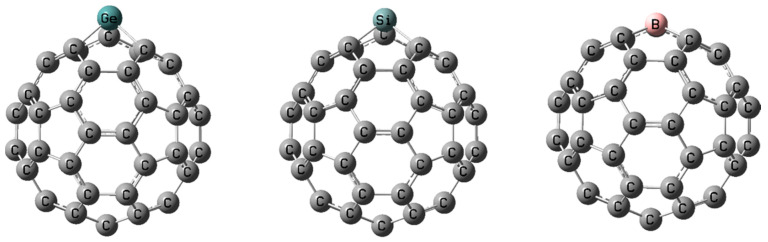
The structures of C_59_Ge, C_59_Si, and C_59_B heterofullerenes [14].

The 1985 discovery of the C_60_ molecule, known as Buckminsterfullerene, represented a crucial event in carbon chemistry and nanoscience. Initially identified by Kroto et al. through mass spectrometric analysis of carbon clusters generated in a laser vaporization apparatus, C_60_ was proposed to possess a truncated icosahedral geometry of remarkable symmetry and stability [1]. Subsequent work by Krätchmer and his collaborators [2,3] provided the first experimental confirmation of the IR and UV spectral features of C_60_, thereby verifying its synthesis in macroscopic quantities through evaporating graphite under high-pressure inert gases, such as He or Ar. As a result of these efforts, solid C_60_ was isolated, allowing for further spectroscopic analysis [2,3]. Since then, fullerenes have become increasingly important as a class of carbon allotropes with unique structural, electronic, and physicochemical properties [4,5,6]. Due to their exceptional thermal stability, delocalized π-systems, and propensity for functionalization of the highly symmetric cage-like C_60_ structure composed entirely of sp^2^-hybridized carbon atoms, these molecules are attractive for applications in photovoltaics, materials science, nanotechnology, and biomedicine [7,8,9,10,11,12]. Heterofullerenes C_60_, structures in which one or more carbon atoms in the C_60_ cage are substituted by heteroatoms, e.g., B, N, O, P, Si, Ge, As, or certain transition metals (Figure 1), represent a versatile class of nanostructures with properties tunable far beyond those of pristine fullerene [13,14,15,16]. The mentioned substitution leads to obtaining structurally and electronically diverse classes of compounds, such as: borafullerenes, azafullerenes, oxafullerenes, phosphafullerenes, silafullerenes, and metallofullerenes, each exhibiting unique reactivity and electronic profiles [16,17,18,19,20].

Substitutional doping has been successfully achieved for a number of heteroatoms using gas-phase synthesis techniques, in particular laser evaporation of graphite targets doped with the element of interest. Experimental confirmation of such heterofullerenes includes species such as C_59_B [21], C_59_N [22], and C_59_Si [23]. For clarity, the level of experimental certainty differs across dopants: nitrogen substitution has been demonstrated by isolation and solid-state characterization (e.g., C_59_N isolated as (C_59_N)_2_), whereas for boron and silicon, the strongest evidence has been obtained in the gas phase. In particular, ion mobility measurements combined with Mass Spectroscopy (MS) show that C_2n−1_Si+ clusters behave as substitutional heterofullerenes, while C_2n_Si+ exhibit exohedral Si; however, this assignment—though compelling—does not yet constitute crystallographic proof. Substitution of carbon atoms in the fullerene scaffold causes local structural distortions resulting from differences in the covalent radii and binding preferences of the dopant atoms. These distortions also lead to significant changes in the electronic structure of the molecule, including narrowing or shifting the HOMO–LUMO gap, the appearance of local frontier orbitals, and the induction of permanent dipole moments [16,24,25]. As a result, heterofullerenes exhibit enhanced reactivity at specific sites, greater polarizability, and the potential for selective chemical functionalization—features of particular interest for the design of nanostructured materials, sensors, and drug delivery systems. Intensive theoretical and experimental studies have demonstrated the remarkable functional potential of heterofullerenes. For instance, boron- and nitrogen-doped systems show significantly enhanced CO_2_ affinity and tunable binding under applied electric fields, suggesting opportunities for reversible gas capture, whereas phosphorus-substitution has likewise been shown to modulate adsorption energies and enable field-dependent sorption selectivity [26,27]. In turn, transition-metal modification introduces catalytic functionality, as exemplified by Ti-decorated fullerenes that catalyze CO_2_ activation and H_2_ dissociation [28], while early alkali-metal doping experiments, such as potassium-modified C_60_, revealed substantial changes in electronic conductivity [29].

In addition to their distinct electronic structure from their parent fullerenes, heterofullerenes offer new opportunities for functionalization and applications in materials science and biomedicine, becoming the subject of intense theoretical and experimental interest [30]. Such modifications allow fine-tuning of molecular orbitals, reactivity, and electronic characteristics [31]. While substitutions with elements such as nitrogen, boron, and phosphorus have been widely explored [21,22,32,33,34,35,36,37,38,39,40], the incorporation of silicon into the fullerene framework (C_60−n_Si_n_) represents a relatively novel and promising direction [41,42], as silicon shares several valence features with carbon but differs significantly in electronegativity, atomic radius, and bonding preferences. This makes Si-doped fullerenes particularly interesting from the perspective of both structural chemistry and functional materials design.

Considering the vast amount of research on fullerenes, this review is intentionally limited to C_60_ analogs, the most extensively studied member of the fullerene family. Systems comprising smaller carbon cages (<60 atoms) or larger analogs (>60 atoms) are beyond the scope of this work. Furthermore, as there is a large number of studies addressing C_60_ doped with various heteroatoms, we focus exclusively on C_60_ derivatives doped with silicon. This selection highlights silicon’s unique ability to modify the fullerene cage shape, electrical structure, and chemical reactivity, which offers particular opportunities for focused theoretical studies and potential technological applications. Whereas numerous published studies have systematically investigated various silicon-doping motifs within C_60_, including replacement of carbon by silicon atoms in the C_60_ lattice (substitutional doping) [43], encapsulation of silicon atoms or clusters inside the C_60_ cage (endohedral doping) [44], and exohedral adsorption (surface decoration) of silicon atoms [45], in this review, the scope is confined to Si-substituted C_60_, encompassing not only the bare substitutional cages but also a broad range of their exohedral derivatives with adsorbed molecules and endohedral complexes with encapsulated species, while pristine C_60_ systems remain out of the scope of this article.

This study focuses on both experimental and theoretical perspectives on silicon-doped C_60_ fullerenes. A preliminary review of the experimental strategies used for their preparation is presented to define the synthetic context. The principal emphasis, however, is placed on theoretical investigations, with particular attention paid to quantum chemical and semiempirical methods, including molecular dynamics simulations. We discuss computational studies of the intrinsic structural and electronic characteristics of Si-doped C_60_, as well as systems involving endohedral encapsulation and exohedral functionalization of such structures. Through a systematic review of the available literature, this work aims to assist researchers in selecting the appropriate computational approaches and to identify the range of structural, electronic, and reactivity-related properties of silicon-doped C_60_ that can be reliably investigated using theoretical calculations.

## 2. Synthesis of Si-Doped C_60_ Fullerenes

Since the discovery of fullerenes, the incorporation of heteroatoms into carbon cages has opened new avenues for tuning their physical and chemical properties. Silicon, isoelectronic with carbon, has garnered significant attention for substitutional, endohedral, and exohedral doping, offering unique electronic properties attributed to its greater size and specific bonding characteristics. Silicon-doped fullerenes are significant in the realm of novel nanomaterials and semiconductor chemistry [42,46,47].

The incorporation of silicon atoms into fullerene cages has been achieved almost exclusively by high-energy gas-phase methods, where extreme temperatures and short reaction times favor substitutional doping during cluster nucleation. Over the course of nearly three decades, experimental approaches to silicon-doped C_60_ systems have evolved to reflect both ongoing issues with dopant control and performance optimization and advancements in fullerene production processes.

Kimura et al. were among the pioneers in employing laser vaporization of graphite rods with low silicon content to synthesize silicon-doped fullerenes. Mass spectrometric analyses indicated that silicon atoms were incorporated into clusters of varying sizes. Only a limited number of SiC_n_ clusters (n = 56–61) were seen in the mass range corresponding to C_60_, indicating that silicon weakens the integrity of the fullerene cage structure, as it possesses a higher atomic radius than carbon, resulting in the distortion of the icosahedral cage structure and limitation of its structural stability [46].

Further evidence for substitutional Si-doping in C_60_ cages was subsequently provided by Fye et al. Using arc discharge between graphite electrodes in an atmosphere enriched with volatile silicon species, the authors generated fullerene-containing soot that was subsequently analyzed by laser desorption mass spectrometry. The detection of both exohedral and substitutional Si-containing species suggested that the high-temperature plasma environment supported a variety of doping processes. However, it was not possible to properly control the spatial distribution of silicon atoms inside the carbon framework, and dopant incorporation per cage was generally poor [48].

**Figure 2:**
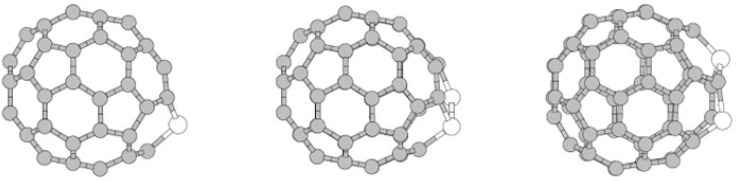
Structure of C_60−n_Si_n_ (n = 1–2) isomers. From the left: C_59_Si, C_58_Si_2_ (ortho), C_58_Si_2_ (meta); white spheres are Si atoms. Adapted from [23], licensed under CC BY 4.0.

Soon after, Cao and collaborators [47] used arc evaporation of graphite electrodes loaded with silicon carbide powder in a helium atmosphere to produce silicon-containing fullerenes in larger quantities. Macroscopic levels of silicon-doped fullerene derivatives were obtained by solvent extraction of fullerene-containing carbon black with carbon disulfide and verified by mass spectrometric characterization. This was the first reported synthesis that yielded amounts sufficient for further chemical and physical characterization, but the mass spectrometric identification did not allow unambiguous discrimination between substitutional incorporation of silicon into the C_60_ cage and exohedral attachment of silicon species on its surface [47]. By contrast, Fye and Jarrold used ion mobility spectrometry (IMS) with MS to separate isomers by collision cross-section and showed that (i) for C_2n−1_Si+, the measured mobilities match silicon-substituted cages; and (ii) for C_2n_Si+, the increased inverse mobilities (≈3–5%) align with an exohedral Si model rather than endohedral or ‘hole-defect’ structures; the water-adduct reactivity and fragmentation channels further support these assignments [48]. In Cao et al., bulk extraction (CS_2_) of Si-containing fractions was achieved, but unambiguous discrimination between substitutional and exohedral geometries was not possible with the reported spectrometric data [47]. Ray et al. advanced the field by employing a graphite–silicon composite arc discharge specifically designed to promote substitutional Si-doping, rather than exohedral functionalization or endohedral encapsulation. Mass spectrometric analysis revealed the presence of heterofullerenes such as C_59_Si and C_58_Si_2_, and further UV–vis, photoelectron, and IR spectroscopy studies showed frontier orbital shifts and vibrational distortions consistent with substitutional incorporation of silicon, providing direct evidence of carbon replacement by silicon in a C_60_ framework (Figure 2). Controlled arc discharge enabled reproducible formation of Si-substituted cages, although obtained in low yields and requiring challenging separation [23]. Pellarin and collaborators investigated another approach based on laser evaporation of graphite–silicon composite targets. Based on time-of-flight mass spectrometry measurements, the authors confirmed that up to nine silicon atoms were added to the C_60_ cage without causing complete structural collapse. According to the researchers, nucleation begins with a mixed Si-C gas phase, and the degree of cage distortion caused by the silicon atoms directly affects the stability of the resulting heterofullerenes [49]. Kimura et al. [46] observed SiCn (n ≈ 56–61) mass peaks in the fullerene-size region using TOF-MS of siliconized graphite targets. The even–odd alternation patterns mirror those of pristine/B-doped series and are consistent with heterofullerene-like clusters; nevertheless, MS alone cannot distinguish substitutional from exohedral Si. Moreover, because C2-loss is a characteristic fragmentation pathway of fullerenes, care is needed not to over-interpret the appearance or depletion of neighboring peaks solely in terms of composition without corroborating structural probes.

Building on these findings, Pellarin et al. used photofragmentation experiments to investigate the stability of silicon-doped heterofullerenes. They prepared substitutionally doped C_60_ derivatives by inducing rearrangements in silicon carbide clusters using laser annealing. Although higher silicon concentrations increased the probability of cage opening, the fragmentation patterns indicated that the doped cages maintained a closed shell structure even at high internal energies. They concluded that cage stability and dopant implantation could be maximized by post-formation heat treatment [49].

In turn, Blase et al. employed photofragmentation mass spectrometry to analyze heterofullerenes derived from laser-vaporized graphite–silicon targets. Moderate doping maintained the integrity of the fullerene framework, whereas elevated substitution levels resulted in increased fragmentation and partial cage opening. Silicon atoms exhibited a tendency to cluster in neighboring pairs, resulting in the elongation of Si–C bonds and the introduction of partial sp^3^ hybridization [50]. At elevated doping levels, alternative square-faced cage architectures have been proposed, potentially accommodating as many as twelve silicon atoms [51].

**Figure 3:**
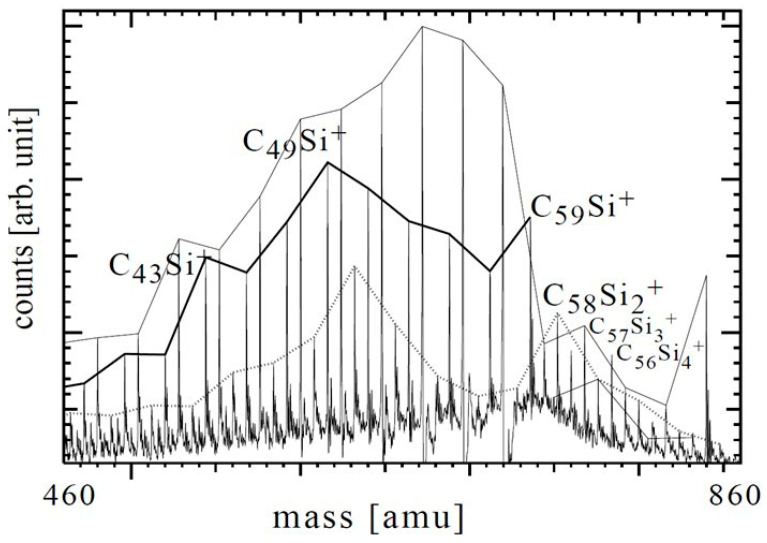
Photoionization mass spectrum of C_60−n_Si_n_ clusters exposed to high laser fluence. Reproduced with permission from SNCSC [52].

In a combined experimental–theoretical study, Billas et al. investigated C_59_Si and C_58_Si_2_ species using mass spectrometry (Figure 3) and Car–Parrinello molecular dynamics simulations. Based on the conducted research, the existence of distinct substitutional heterofullerenes was verified, while simulations showed that silicon atoms induce localized distortions in the sp^2^ carbon framework without disrupting the overall cage integrity. Importantly, the computations showed a preference for adjacent silicon–silicon configurations, similar to the bonding observed in disilabenzene, which may have implications for the design of multi-doped fullerene systems [52].

A significant advance in this field was reported by Ohara and collaborators, who achieved high-yield synthesis of C_58_Si_2_ via arc discharge under carefully controlled doping ratios. The silicon atoms preferentially occupy nearby locations in the carbon network, which is consistent with previous fragmentation studies and theoretical predictions, according to the structural analysis of the products after they were extracted by solvent extraction. This study showed that yield and structural selectivity can be improved by precisely adjusting the doping environment [53].

In turn, in experiments performed by Bulina et al., the first atmospheric-pressure arc synthesis of C_58_Si_2_ going beyond traditional vacuum techniques was achieved. The method discussed involved adding fine silicon powder to carbon-helium plasma and then processing the resulting soot through a series of Soxhlet extractions. The presence of compounds with a fullerene-like structure, with clustered silicon atoms incorporated into a cage, was confirmed by mass spectrometry data. Although control of impurity content remained challenging, the atmospheric-pressure method simplified the equipment and was able to provide a more scalable route [54].

Collectively, these investigations illustrate the diversity of experimental approaches to synthesizing silicon-doped C_60_ and emphasize the link between synthesis conditions, structural stability, and doping efficacy. However, precise control of dopant number, location, and distribution remain difficult, as silicon tends to substitute locally and cluster within the cage, altering electronic and vibrational properties. Traditional methods, such as arc-discharge of SiC-doped graphite, yield only trace amounts of Si-fullerenes, with most products being pristine cages and minor Si-containing derivatives that require laborious separation and sensitive analysis due to their low stability [52,54].

Efforts to improve silicon incorporation involved further refinements to synthesis, including plasma-assisted environments and enhanced laser vaporization, aiming to boost the reactivity between silicon and carbon fragments under inert atmosphere and elevated temperatures. Nevertheless, analysis of the resulting mixtures revealed products consisting mainly of exohedral silicon-decorated fullerenes, various non-cage Si–C clusters, and only minor fractions of substitutional Si-fullerenes, greatly complicating their identification and purification [47,54].

Unambiguous evidence for substitutional Si-doping has been provided by mass spectrometry, which detected fullerene-like clusters such as C_59_Si^+^ with the expected mass and fragmentation patterns [46,48,52,54]. However, the isolation of pure Si-doped fullerenes remains extremely challenging, as their high reactivity and intrinsic instability lead to rapid decomposition or polymerization during extraction and purification, underscoring the need for improved synthesis and isolation methods, and a deeper understanding of how atomic size and electronic effects govern cage stability and doping selectivity (Table 1).

## 3. Theoretical Investigations of Si-Doped C_60_ Fullerenes

Experimental efforts have long been directed toward exploring the synthesis and properties of Si-doped C_60_. Although these studies have provided early evidence supporting the formation and characterization of silicon-containing fullerene structures, it is the theoretical investigations that have offered crucial insights into the stability, electronic structure, and potential reactivity of these novel nanostructures [50,51,52]. Computational investigations of Si-doped C_60_ span a hierarchy of methods. The chosen level of theory is essential for accurately depicting the effects of silicon integration, including changes in frontier orbital energies, predicted reactivity, and subtle distortions in cage geometry. In this context, semiempirical approaches (PM3, MNDO, SCC-DFTB) have been applied for rapid screening of candidate geometries and stability trends, often as a prelude to higher-level studies [42,55,56,57,58,59,60,61]. In turn, density functional theory (DFT), particularly with hybrid and dispersion-corrected functionals, provides reliable insights into cage deformation, charge redistribution, and HOMO–LUMO modulation, and remains the dominant tool for structural and reactivity studies of Si-doped heterofullerenes and related derivatives [52,62,63,64,65,66,67,68,69,70,71,72,73,74,75]. Molecular dynamics simulations—ranging from SCC-DFTB trajectories to first-principles Car–Parrinello dynamics—are often used to elucidate finite-temperature stability, fragmentation pathways, and substitutional versus exohedral stability of Si-containing cages [43,45,52,58,59,61,66,70,76,77,78,79]. Ab initio computations of fullerenes are relatively rare due to the computational cost of correlated wavefunction methods. Some HF and MP2 studies provided benchmarks for C_60_ and smaller fullerene cages [80,81,82,83], later extended by coupled-cluster analyses of electronic correlation [35,83,84,85]. Overall, ab initio approaches have remained largely confined to small cages and endohedral systems, whereas DFT has emerged as the dominant framework for larger fullerenes. To the best of our knowledge, only a single HF study has been reported for Si-doped C_60_, focused on C_59_Si and its Ge and Sn analogs [86].

To facilitate a systematic discussion, the part of this review devoted to computational studies is organized into three subsections: (i) substitutional Si-doped C_60_, referring to systems in which carbon atoms of the fullerene cage are replaced by silicon atoms; (ii) exohedral complexes of Si-doped C_60_, encompassing cases where additional atoms or molecules are adsorbed onto the surface of the silicon-substituted cage; and (iii) endohedral complexes of Si-doped C_60_, where atoms or small clusters are encapsulated within the Si-doped fullerene cavity.

### 3.1. Substitutional Si-Doped C_60_ Fullerenes

Across representative isomers, C–Si bond lengths fall in ≈1.83–1.98 Å, with Si–Si separations ≈2.30–2.66 Å (cf. Table 2 in Koponen-type datasets); these values exceed typical C–C distances and rationalize local cage dilation. Aromaticity indicators at the cage center change modestly—for example, NICS(0) for C_59_Si is ≈−2.80 versus ≈−2.72 for C_60_—while HOMO–LUMO gaps generally narrow, and dipole moments increase due to charge localization near the Si site. We also note the systematic growth of visible/near-IR absorption with higher Si content and the reduced isomer specificity at heavy doping, consistent with prior TD-DFT/MD analyses. Substitutional doping of C_60_ with silicon, accomplished by substituting one or more carbon atoms in the fullerene structure with silicon atoms, is a widely investigated modification approach for heterofullerenes. It directly modifies the highly symmetric π-conjugated structure of pristine C_60_, consequently affecting its electronic distribution, structural stability, and chemical reactivity—features that are often analyzed using theoretical methods. Numerous theoretical studies on substitutional Si-doping have elucidated the influence of dopant quantity and spatial distribution on cage deformation, HOMO–LUMO properties, charge localization, and adsorption characteristics, thereby positioning this class of systems as fundamental to the comprehension of Si–C heterofullerene chemistry. Among this group of derivatives, the most frequently studied one is the C_59_Si derivative, in which only one carbon atom has been replaced by silicon [55,57,64,65,68,72]; however, calculations of structures with two to thirty silicon atoms can also be found [42,52,56,66,67,69,70,71,73,76,77,78,79]. Analysis of the surveyed literature indicates that the most commonly employed computational packages were Gaussian (versions 98, 03, 09) [42,55,56,64,65,67,68,70,72], DFTB+ code (Density Functional Tight-Binding code) [73], SIESTA code [66,69,71], VASP code (Vienna Ab initio Simulation Package) [73], DMol3 [56], MOPAC (within the CAChe software suite) [57], Dalton 2013 [64], OCTOPUS 2.0.1. [66], CPMD code (Car–Parrinello Molecular Dynamics) [52,70,76,77,78,79,87]. A survey of computational studies on substitutional Si-doped C_60_ shows a clear predominance of density functional theory (DFT), with hybrid (B3LYP) and generalized gradient approximation (GGA) functionals such as PBE and BLYP frequently applied in combination with split-valence polarized basis sets (e.g., 6-31G(d), 6-311G(d)) [42,56,64]. B3LYP/6-31G(d) emerged as the standard choice for geometry optimization and electronic structure analysis, providing a practical balance between computational cost and accuracy [65,67,72]. Some works introduce the larger and more flexible 6-311G(d) basis set in order to achieve improved accuracy in structural and magnetic characteristics, particularly for NMR and NICS calculations [42,67,70]. For absorption spectra and excited state properties, methods based on time-dependent DFT (TDDFT) with functionals such as TD-LDA and TD-BLYP, and smaller basis sets like 3-21+G(d), are often preferred as they make it possible to strike a balance between accuracy and computational cost [64]. Less commonly, alternative functionals (BP86, BPW91, CAM-B3LYP) and basis sets (cc-pVDZ, aug-cc-pVDZ, DNP) are used to test their effects on transition energies or for benchmarking purposes [56,64,68]. Overall, the B3LYP/6-31G(d) combination dominates in ground-state computations, while TDDFT approaches with LDA- or GGA-type functionals and medium-sized basis sets are typical for optical property studies.

The most frequently performed calculations for the discussed structures were geometry optimizations [42,55,57,64,65,66,67,68,69,70,71,72,73,77,78,79,87] and electronic structure and properties analysis (ESPA) of previously optimized structures, such as, e.g., molecular orbital energies (HOMO/LUMO), energy gaps, charge distribution analyses (such as Mulliken population or electrostatic potential mapping), spatial localization of electron density, atomic charges, and dipole moments [42,55,57,64,65,66,68,69,71,72,73,77,78,79,87]. The ESPA calculations for substitutional Si-doped fullerenes consistently revealed characteristic modifications relative to pristine C_60_. Substitution of carbon atoms by silicon typically resulted in electron density localization on the dopant sites, accompanied by charge transfer to adjacent carbons. These effects were reflected in reduced HOMO–LUMO gaps, enhanced dipole moments, and pronounced alterations in local atomic charges and overall molecular polarity. Such electronic reorganizations, arising from the spatial localization of electronic states around silicon atoms, influence both the stability and reactivity (decreasing the stability and increasing the reactivity) of the heterofullerenes. Regarding NICS calculations, it has been shown that the NICS values at the cage center of Si-substituted fullerenes (C_60−n_Si_n_) were typically less negative than those for pristine C_60_, indicating a reduction in aromaticity as the silicon content increases [65,67,72]. An interesting application of computational methods were made by Koponen et al. and Lan et al.; the authors used the TDDFT (Time-Dependent Density Functional Theory) method in order to compute photoabsorption spectra to assess spectral changes upon increasing silicon content or solvent influences, respectively [64,66]. Several studies utilized first-principles molecular dynamics (FPMD) to investigate the structural stability and finite-temperature behavior of highly silicon-doped fullerenes. These simulations enabled the identification of fragmentation mechanisms, the role of atomic segregation (Si/C subnetworks), and the impact of charge and temperature on cage stability, providing insights necessary for predicting the synthetic feasibility and chemical reactivity of these novel nanomaterials [76,77,78,79,87]. The semiempirical methods were mainly used for fast, broad surveys of structural possibilities and initial screening, whereas DFT provided reliable, quantitative insights into energetics, electronic properties, spectroscopy, and thermal stability of substitutional Si-doped C_60_ fullerenes, as this two-tiered approach efficiently balances computational efficiency with the need for chemical accuracy. The analyzed studies aimed to determine how silicon substitution alters the cage geometry, electronic structure (e.g., charge distribution, band gaps), dynamical stability at finite temperature, and optical responses, ultimately identifying stability limits and unique features of Si-doped fullerene-based nanomaterials in neutral and charged states. The most interesting applications of calculations in the study of these structures are presented below in more detail; Table 3. provides a summary of the computational protocols applied, specifying the semiempirical methods, DFT functionals, and basis sets used in the respective studies on substitutional Si-doped C_60_ fullerenes.

The semiempirical (PM3) calculations of the structural and electronic properties of the substitutional heterofullerene C_59_Si, were the topic of a study by Lu et al. [55]. The authors focused on optimizing possible isomers and analyzing their stability, local bonding, and electronic structure, with particular attention to the localization of electronic states near the Si atom. Their calculations revealed that the incorporation of silicon into the C_60_ framework induces significant changes in the local electronic density, resulting in partial charge transfer from the silicon to the carbon cage, and promoting the formation of a chemically distinct site on the fullerene surface. The electronic structure analysis indicated the localization of the frontier molecular orbitals, especially the highest occupied molecular orbital (HOMO), around the Si atom, which may enhance the chemical reactivity of the doped site. These findings offer insight into the microscopic modifications introduced by light heteroatom doping in fullerene systems, which could be relevant for tuning their electronic and chemical properties [55].

In turn, Ibrahim and collaborators used the semiempirical PM3 method to model C_60_ doped with silicon, germanium, and aluminum. Doping with silicon resulted in changes in bond lengths, molecular dimensions, charge distribution, and dipole moment similar to those observed with germanium and aluminum, but the calculated data indicated that the specific magnitude of these effects and the total dipole moment differ slightly for each dopant, reflecting element-dependent variations in the modification of C_60_’s physical properties [57].

Other applications of semiempirical methods are offered by Fan et al. [56] and Guirado-López [42]. In the first of the discussed papers, a semiempirical method—specifically, the modified neglect of differential overlap (MNDO)—was initially used to generate and optimize the molecular structures of Si-doped fullerenes. The MNDO-optimized geometries were then subjected to DFT calculations for further refinement of the electronic properties and relative energies. The semiempirical approach allowed rapid screening and initial relaxation of a vast number of possible Si/C arrangements, serving as a computationally efficient precursor to more demanding first-principles calculations, and thus enabling the effective exploration of the configurational space for low-energy isomers in the extensive C_60−n_Si_n_ system [56]. In studies by Guirado-López, the electronic structures and stabilities of endohedral, exohedral, and substitutionally Si-doped C_60_ were compared to Si-doped C_70_ using both MNDO and DFT calculations. The results showed that substitutional doping is energetically most favorable, with endohedral and exohedral arrangements being less stable, and that Si doping generally induces localized structural distortions and charge transfer from Si to C. These modifications were reflected in notable changes to the electronic spectra, stability, and chemical reactivity of the doped fullerenes, compared to pure carbon cages [42].

In a set of studies conducted by Matsubara and collaborators, the authors used advanced computational methods, primarily DFT and FPMD, to elucidate the structural and electronic properties of highly Si-doped fullerenes (n = 12, 20, 24, 30). Their results demonstrate that stable cage-like structures can still be formed at extreme doping levels, provided that silicon atoms aggregate into contiguous subnetworks while carbon maintains locally conjugated domains. The structural models reveal pronounced segregation of Si- and C-rich regions, with the fullerene framework expanding anisotropically to accommodate the larger Si atoms. These calculations established that substitutional Si incorporation does not simply destroy cage integrity but instead drives a reorganization into hybrid, fullerene-like nanostructures with distinct electronic and geometric properties [87]. A key finding was the identification of a doping threshold (n ≈ 20 for C_60−n_Si_n_), above which the inner Si atoms drive thermal instability and fragmentation. The studies revealed that structural stability inferred from 0 K optimizations is insufficient for predicting real-world, finite-temperature behavior, particularly as segregated isomers may fragment thermally while non-segregated ones persist [76]. Furthermore, electronic and chemical properties can be tuned by charging the clusters, with anions potentially being more reactive due to enhanced negative charge within the inner Si region. This portfolio of research deepens the mechanistic understanding of Si-doping in fullerenes and sets the stage for experimental realization and application of these novel nanostructures [43,70,76,77,78,79,87].

**Figure 4:**
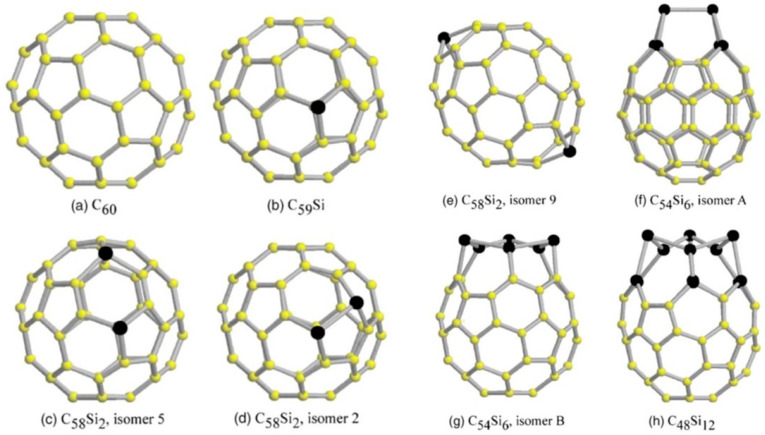
Structures of the Si-doped C_60_ isomers. yellow: carbon, black: silicon. Used with permission from [66]; permission conveyed through the Copyright Clearance Center, Inc., Danvers, MA, USA.

Koponen et al. elucidated key aspects of the structural changes occurring in substitutional Si-doped C_60_ with increasing silicon content. The results of performed computations showed that silicon atoms tend to aggregate within the fullerene cage, introducing significant geometric distortions as the number of silicon dopants increases (Figure 4). The authors concluded that increasing the degree of silicon substitution in C_60_ is accompanied by the persistence of long Si–Si bonds (~2.4 Å), significantly exceeding the average C–C bond length (~1.5 Å) and reflecting the distinct bonding character of silicon within the fullerene framework (Table 2). These structural changes disrupt molecular symmetry and lead to notable modifications in the electronic absorption spectra, such as enhanced absorption in the visible and near-infrared regions as more silicon is incorporated into the cage [66].

Bai et al. utilized the DFT method to analyze the structure, stability, electronic properties, infrared spectra, dielectric constants, and aromaticity of C_59_Si and compared it to B-, N-, Al-, P-, Ga-, Ge-, and As-counterparts. The results showed that substitution leads to varying degrees of cage distortion, modulates electronic properties, generally narrows the HOMO–LUMO gap, increases dielectric constants, and typically enhances aromaticity relative to C_60_, but with no direct correlation between aromaticity and sphericity. Compared to other doped C_60_, C_59_Si showed notable cage distortion, but this is part of a continuous trend where increasing heteroatom size leads to greater cage asphericity and a greater sphericity parameter (SP). Si falls into an intermediate group with Al and P (SP ≈ 1.2), not as close to the sphere as B, N (SP ≈ 0.1), and less distorted than Ga, Ge, As (SP > 3). The NICS value for C_59_Si (−2.80) was calculated as slightly more negative than pristine C_60_ (−2.72), indicating a marginally greater aromaticity, similar to C_59_Ge (−3.18) and less negative than C_59_B (−5.94); this is consistent with group trends [65].

Studies by Lan et al. [64] and Koponen et al. [66] utilized advanced TDDFT approaches to explore how silicon substitution in fullerenes affects their optical absorption properties. They consistently demonstrate that a single or a few silicon atoms introduce new or enhanced absorption features, notably in nonlinear optical (TPA) or lower-energy regions, differentiating isomers especially at low doping levels. While heavily doped fullerenes lose isomer specificity in their spectra, both works highlight the increased sensitivity of TPA [64] and broad absorption in the visible/near-IR [66], establishing optical spectroscopy—and especially nonlinear methods—as valuable for distinguishing silicon-doped fullerene isomers.

In summary, studies on substitutional Si-doped C_60_ have demonstrated that substitution of C_60_ with silicon leads to significant local electronic and structural changes, including charge transfer and orbital localization around the Si atom, enhancing the chemical reactivity of the doped site and tuning fullerene properties. Moreover, semiempirical and DFT studies consistently showed that substitutional doping is energetically favored over endohedral and exohedral Si-doping, producing element-specific effects on molecular distortion, stability, and electronic spectra. At higher Si concentrations, spatial segregation becomes energetically preferred, but excessive Si content induces thermal instability, with the overall optical, electronic, and aromatic properties of C_59_Si following systematic trends across group IV/V heterofullerenes and exhibiting distinctive nonlinear optical absorption features critical for isomer differentiation.

### 3.2. Exohedral Complexes of Substitutional Si-Doped C_60_ Fullerenes

Exohedral complexes of substitutionally Si-doped C_60_ represent a distinctive class of hybrid nanostructures in which external atoms or molecules are adsorbed onto the surface of the silicon-substituted cage. The presence of Si within the carbon framework significantly alters the electronic density distribution, frontier molecular orbitals, and local reactivity of the cage, thereby enhancing its affinity toward adsorbates. Compared with pristine C_60_, Si-doped systems often exhibit stronger adsorption energies, increased charge transfer, and modified band gaps, which render them attractive candidates for applications in sensing, catalysis, and drug delivery. In particular, the exohedral binding of pharmacologically active compounds to Si-doped cages has been proposed as a means to improve drug loading, stability, and controlled release.

Computational investigations on exohedral complexes of substitutional Si-doped C_60_ fullerenes span a wide range of substitutional derivatives, from monosilicon-substituted cages (C_59_Si) to double-doped systems (C_58_Si_2_) and heavily doped forms such as C_60−m_Si_m_ with m ≥ 6 (Table 4.). Early studies of structural stability and fragmentation applied tight-binding molecular dynamics, showing that substitutional Si atoms stabilize the cage more efficiently than exohedral Si clusters, which tend to segregate on the surface [45,60,61]. Subsequent DFT-based works extended the scope to highly substituted fullerenes, including configurations with Si dimers at pentagon–pentagon junctions [88], and to Janus-type assemblies, where Si substitution sites served as nucleation centers for Si_10-20_ or silicide clusters [89]. These studies demonstrated how the number and position of dopants critically control electronic density, thermal robustness, and further functionalization pathways.

The adsorbates investigated in Si-doped C_60_ exohedral complexes fall into six main categories. The first group are silicon atoms and Si_n_ clusters that were modeled as external guests, revealing anisotropic electronic polarization and cluster formation [45,59,60,61,89]. Secondly, ionic adsorption was studied for alkali and alkaline earth cations (Li^+^, Na^+^, K^+^, Be^2+^, Mg^2+^, Ca^2+^), showing enhanced affinity and stronger band gap modulation for Si-doped cages compared to pristine C_60_ [90,91]. In the third group of studied objects, one can find small inorganic molecules, including hydrogen [58], hydrogen sulfide [92,93], sulfur dioxide [93], carbon monoxide [94], and oxygen [94,95], highlighting the catalytic and storage potential of Si-doped C_60_ fullerenes. The fourth group consists of numerous organic molecules, pollutants and ligands, including TNT [96], hydroquinone [97], 4-phenylpyridine [98], β-propiolactone [99], thiophene [93], PPTO [100], TFMPP [101], and acrolein [102] or hexachlorobenzene [103]. The fifth set are biologically relevant small molecules, such as amino acids, namely phenylalanine [104], serine [105], cysteine [106], and biogenic amines, i.e., histamine [107], tyramine [108], and epinephrine [109]. The sixth group contains a collection of studies addressing pharmacologically active compounds used as drugs, such as carbamazepine [24], 5-fluorouracil [75], amphetamine [74], amantadine [62,110], favipiravir [63], molnupiravir [111], paracetamol [112,113], metronidazole [114], ornidazole [25], valproic acid [115], and NSAIDs such as diclofenac, mefenamic acid, and nimesulide [116], as well as methadone [14], ifosfamide [117], and epigallocatechin gallate [118]. In nearly all cases, the Si site acted as the preferential binding center, enabling stronger chemisorption, larger charge transfer, and more pronounced electronic sensitivity than pristine fullerenes.

In terms of computational protocols, nearly all studies employed geometry optimizations followed by electronic structure and property analysis (ESPA). The ESPA typically encompassed HOMO–LUMO gap analysis, DOS calculations, NBO charge transfer, and reactivity descriptors such as hardness, softness, electrophilicity, and ΔN_max_ [74,75,94]. Advanced wavefunction analyses, including QTAIM, RDG, and NCI, were increasingly applied to probe bonding characteristics and noncovalent interactions [24,110,111]. In several studies, adsorption energies (E_ads_) were obtained with BSSE corrections applied [24,74,75,88,90,94,97,99,107,108,115,116,117,118,119,120]. In many cases, solvent effects were modeled through PCM or SMD [24,62,63,75,93,94,97,99,100,107,109,111,112,115,116,117,119,121]. TD-DFT was used to predict UV-Vis spectra in sensor design for methadone [14], while vibrational, NMR, and even nonlinear optical (NLO) responses were explored for selected drug complexes [62,104,111,115,116,117,119].

With respect to methodology, DFT dominated, though semiempirical methods were also used. Semiempirical approaches, particularly SCC-DFTB, were employed in selected studies, including hydrogen storage simulations [58], high-temperature dynamics of Si incorporation into fullerene cages [59], and thermal fragmentation analyses via tight-binding MD [61]. Among DFT functionals, B3LYP was most widely applied [14,25,62,75,90,92,95,96,97,98,99,100,101,104,108,109,111,112,114,115,116,117,119,121], while dispersion-corrected variants such as B3LYP-D3 and range-separated hybrids ωB97X-D were also employed [24,74,102,103,105,106,107,110,117,118,120]. The meta-hybrids M06-2X and M06L were applied in drug delivery and sensing studies [14,24,63,75,88,97,98,102,103,104,105,106,110,118,121], while GGA functionals including PBE [91] and PW91 [89] were used mainly in plane-wave frameworks. The basis sets included: a typical split-valence double-ζ basis set with polarization functions on heavy atoms—6-31G(d) [14,24,25,59,62,63,75,90,92,94,96,97,98,99,100,104,112,116,117,118,119,120,121], a triple-ζ basis—6-311G(d) [102,103,104,105,106,110], a basis with additional diffuse functions—6-31+G, 6-311+ or 6-311++ [74,88,93,95], a basis with additional polarization function p-type on hydrogen atoms—6-31G(d,p) [107,108,109,114], and the correlation-consistent cc-pVDZ [62,97,101,111,115], depending on the property targeted. The most frequently used computational packages were Gaussian (versions 09, 16) [24,25,62,63,92,93,94,95,96,97,98,99,101,104,105,109,110,111,112,114,115,116,117,118,119,121] and GAMESS [14,74,75,88,90,120], but less popular computational software such as VASP [89,91] and SIESTA [58] was also utilized.

Computational studies on exohedral decorated Si-doped C_60_ have been strongly application-driven, aiming to link molecular decoration with functional performance. The performed calculations showed that Si incorporation can enhance drug delivery, improve aqueous stability, and generate spectroscopic fingerprints [25,62,75,113,114,115,117]. The mentioned computations consistently predicted strong and often chemisorptive interactions with therapeutics ranging from antivirals and antibiotics to amino acids, with double-Si doping even enabling co-delivery strategies with flurbiprofen and salicylic acid [119]. Moreover, computational analyses identified SiC_59_ as a particularly effective platform, showing enhanced adsorption energies, greater charge transfer, and sharper band gap modulations than pristine C_60_ [14,24,74,97,98,99,101,107,108,109,118]. Simulations further highlighted sensitivity to environmental and inorganic targets, including H_2_S, SO_2_, thiophene, and alkali/alkaline earth cations, where Si doping improved selectivity and response [90,92,93]. Energy and catalysis applications were likewise explored through modeling, which revealed lower lithium-ion migration barriers in doped cages [91], stable multi-H_2_ adsorption enhancing the storage capacity [58], and higher catalytic activity for oxygen reduction and CO oxidation [94,95].

At a more fundamental level, simulations provided mechanistic insights into how silicon affects fullerene stability and reactivity. Comparative modeling distinguished substitutional from exohedral doping in terms of fragmentation and clustering [45,60,61], revealed Si-rich pentagon junctions as cycloaddition hotspots [88], and demonstrated nucleation of Janus-type nanostructures [89]. Crucially, non-equilibrium molecular dynamics indicated that true substitutional incorporation requires simultaneous carbon removal, while simple Si supply results only in exohedral clustering [59].

**Figure 5:**
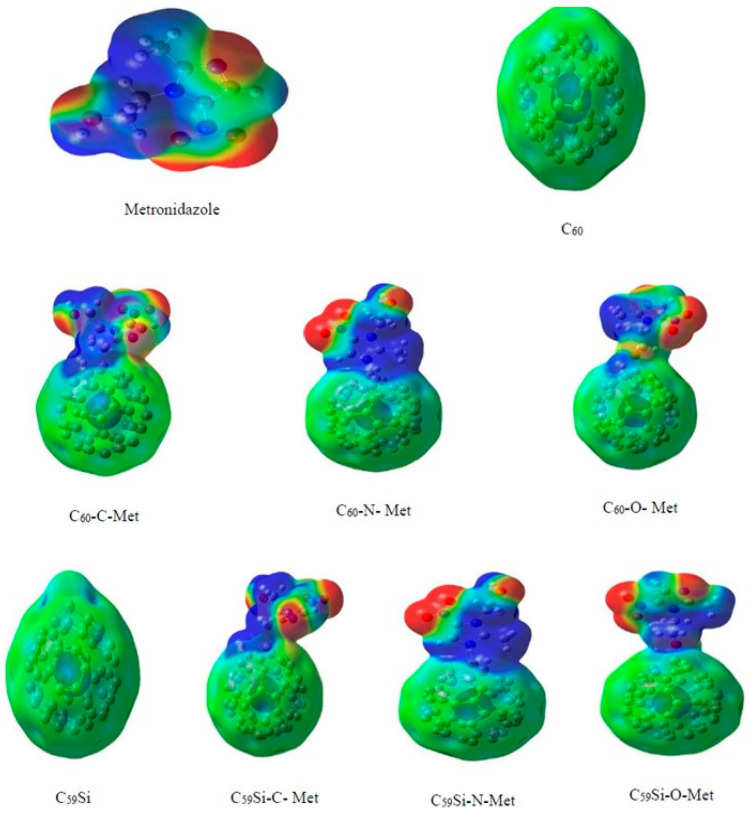
The MEP images of metronidazole and C_59_Si as drug-nanocarrier. Adapted from [114], licensed under CC BY 4.0.

This general review provides the necessary context for a more detailed examination of the most interesting computational studies that clearly characterize the importance of theoretical studies on adsorption in exohedral complexes. Hazrati and collaborators established an early benchmark by showing that substitutional doping markedly strengthens the interaction of 5-fluorouracil with C_60_, shifting the binding from weak physisorption in pristine cages to clear chemisorption. Among the doped systems, Si- and Al-substituted fullerenes exhibited the most pronounced stabilization, with significantly more negative binding energies and enhanced charge transfer at the drug–nanocarrier interface. These effects were accompanied by notable reductions in the HOMO–LUMO gap, underscoring the potential of doped fullerenes as efficient and electronically responsive drug carriers [75]. This theme was developed further by Fekri et al. in studies of the interaction of metronidazole and ornidazole with heterofullerenes, clarifying the role of substitutional Si-, Al-, and B-doping of C_60_ in drug delivery. The adsorption occurred predominantly through the oxygen and nitrogen atoms of the drug molecules, with O-containing groups (carbonyl and nitro moieties) and N centers acting as primary binding sites to the dopant atom of the fullerene cage. Comparative analyses demonstrated that Si-doped fullerenes consistently provided stronger binding and larger reductions in the HOMO–LUMO gap than B-doped analogs, while Al-doped systems often exhibited the highest adsorption energies, but with less favorable electronic sensitivity. Overall, the results highlighted that Si substitution offers an optimal balance between binding strength, charge transfer, and electronic modulation, making Si-doped C_60_ particularly promising for nanocarrier applications relative to its B- and Al-doped counterparts [25,114]. The MEP maps revealed that substitutional silicon creates highly localized electropositive regions on the fullerene surface, enabling strong interactions with the electronegative sites of drug molecules (Figure 5).

**Figure 6:**
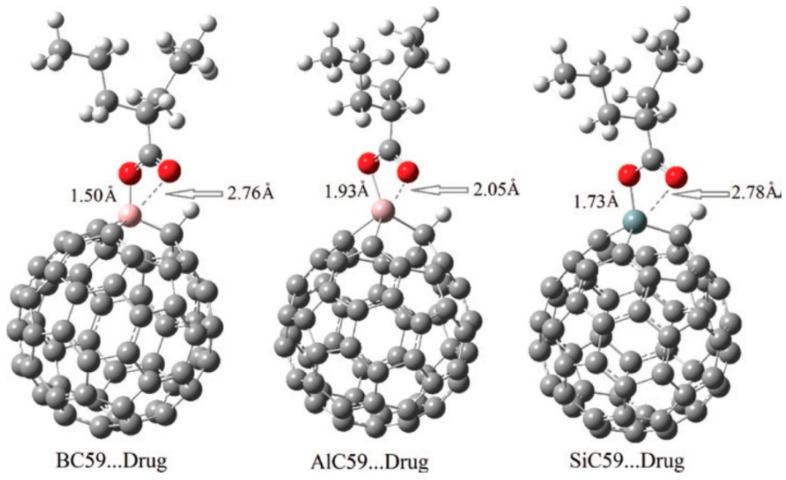
Optimized structures of the BC_59_, AlC_59_ and SiC_59_ complexes with valproic acid. Adapted from [115], licensed under CC BY-NC-ND 3.0.

In turn, studies by Alver et al. reported that SiC_59_ forms stable complexes with valproic acid and piperazine-2,3,5,6-tetraone, combining strong binding with marked band gap reductions. In the case of valproic acid, adsorption occurred predominantly via the carbonyl O atom (Figure 6), with SiC_59_ exhibiting the strongest interaction (E_ads_ ≈ −56 kcal mol^−1^), AlC_59_ showing slightly weaker binding, and BC_59_ providing only moderate stabilization. Notably, while B-doping enhanced interaction energies relative to pristine C_60_, the electronic reactivity and spectroscopic response were far less pronounced than in Si- or Al-doped systems, underscoring the superior ability of Si substitution to combine strong adsorption with significant electronic modulation [100,115].

**Figure 7:**
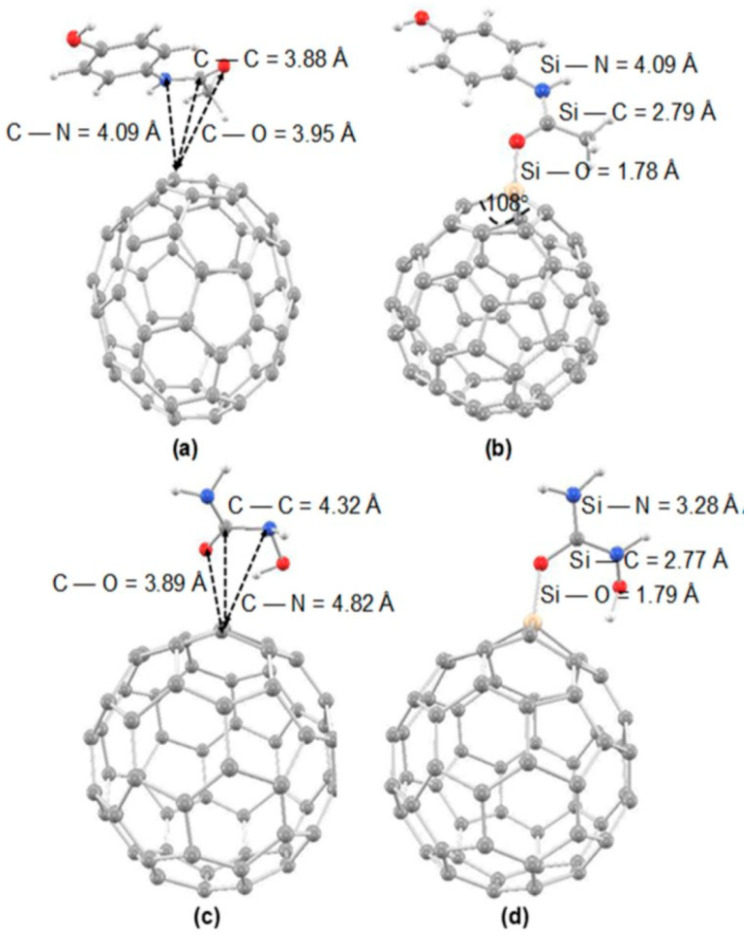
Optimized geometries of (**a**) pure C_60_ with paracetamol, (**b**) Si-doped fullerene (SiC_59_) with paracetamol, (**c**) pure C_60_ with hydroxyurea, and (**d**) Si-doped fullerene (SiC_59_) with hydroxyurea. Adapted from [113], licensed under CC BY 4.0.

Similarly, the calculation performed by Apriati and collaborators showed that SiC_59_ forms particularly stable adsorption complexes with paracetamol and hydroxyurea, with BSSE-corrected binding energies of about −23 to −25 kcal/mol and significant HOMO–LUMO gap reductions from 2.6 eV (for pristine C_60_) to 1.9 eV, indicating enhanced reactivity and stronger electronic coupling upon Si substitution. The active interaction between the attractive site from drug molecules and the Si-doped fullerene results in a shortened distance between drug molecules and the fullerene. The distance between O and Si atoms in the SiC_59_–paracetamol and SiC_59_–hydroxyurea systems decrease almost twice compared with the C_60_–drug complexes (Figure 7) [113].

Çatal et al. extended this concept by modeling double-silicon-doped C_60_ (Si_2_C_58_) as a dual drug carrier, demonstrating selective and simultaneous binding of flurbiprofen and salicylic acid at the Si sites. QTAIM and vibrational analyses confirmed that both flurbiprofen and salicylic acid interact strongly with Si_2_C_58_ through Si–O contacts (1.79–1.81 Å), with BSSE-corrected binding energies of −17 to −19 kcal/mol and pronounced shifts in IR bands (up to ~687 cm^−1^), providing spectroscopic evidence of selective and partially covalent drug–Si_2_C_58_ cage binding. In turn, Kolsuz and collaborators compared co-doping with B, Al, and Si for the delivery of ifosfamide, highlighting how the nature of the dopant dictates both binding energetics and release dynamics. B-doped sites facilitated relatively weak but readily reversible interactions (Eb ≈ −0.5 to −1.2 eV), ensuring rapid drug desorption, though at the cost of stability. By contrast, Al substitution promoted very strong adsorption (Eb ≈ −2.2 eV) accompanied by exceptionally long recovery times (τ > 10^25^ s), effectively immobilizing the drug and impeding controlled release. Si doping was found to offer an intermediate regime, with interaction strengths in the range of −1.3 to −2.1 eV and moderate recovery times, thus achieving the most favorable balance between stability and reversibility. The NBO charge distribution illustrates pronounced electron density shifts upon ifosfamide binding, with increased positive charge on Si and B dopants (from 1.263 to 1.993 a.u. for Si and 0.644 to 0.663 a.u. for B), while Al shows a slight decrease (1.778 → 1.723 a.u.), indicating distinct donor–acceptor roles of the dopant sites within the fullerene cage. This tunable behavior positions Si sites as particularly promising candidates for selective and controllable drug delivery within heteroatom-doped fullerene systems [117].

Within this context, the research program led by Parlak and collaborators provided one of the most systematic and comprehensive series of computational studies. Over nearly a decade, this team demonstrated that Si-doping consistently transforms weak, non-specific interactions of C_60_ into strong chemisorptive binding accompanied by substantial charge transfer, HOMO–LUMO gap reductions, and distinct spectroscopic signatures [62,63,96,98,99,101,104,111,112,121]. For instance, favipiravir and molnupiravir adsorbed strongly onto SiC_59_, with binding energies reaching −43 to −44 kcal·mol^−1^ and concomitant gap reductions of ~12–13%, while diagnostic IR bands exhibited pronounced red-shifts, reflecting the covalent character of the drug–cage interaction [63,111]. Researchers extended these findings to 4-phenylpyridine, showing that pristine C_60_ interacts only weakly (Eb ≈ −0.3 to −7 kcal·mol^−1^), whereas Si-doping enhanced adsorption up to −42.7 kcal·mol^−1^ in the gas phase and −50.3 kcal·mol^−1^ in water, mediated by donor–acceptor interactions between the nitrogen atom of the ligand and the substitutional Si site [98]. Their further studies demonstrated that amino acids such as phenylalanine bind much more strongly to SiC_59_ than to pristine C_60_, with adsorption energies up to −48.7 kcal·mol^−1^ at the NH_2_ group in aqueous medium, underscoring the relevance of Si-doped cages as nanocarriers for biomolecules [104]. Complementary results of this investigation of adsorption ability of Si-doped C_60_ confirmed that SiC_59_ exhibits enhanced sensor sensitivity toward psychoactive ligands such as TFMPP (1-(3-trifluoromethylphenyl)piperazine), where adsorption was accompanied by pronounced band gap reductions and charge redistribution. Collectively, these studies establish that SiC_59_ functions as a versatile nanocarrier and sensor platform for both pharmaceutical agents and organic ligands, with its unique electronic reactivity and spectroscopic fingerprints consistently outperforming pristine C_60_.

**Figure 8:**
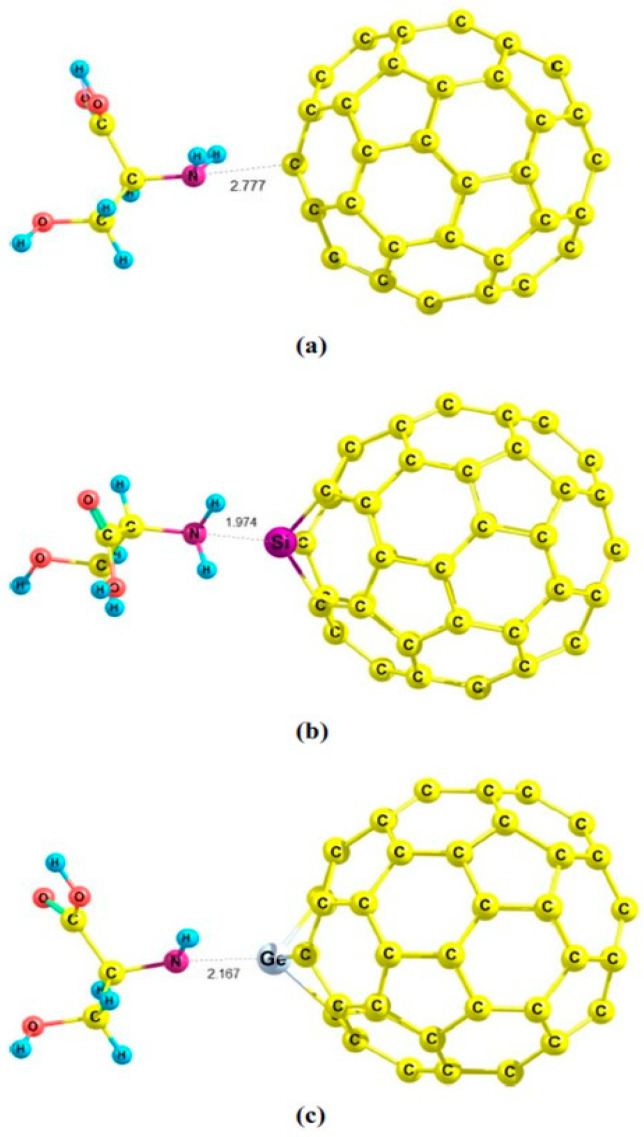
The most stable form of (**a**) serine-C_60_, (**b**) serine-C_59_Si, and (**c**) serine-C_59_Ge. All clusters were optimized using the B3LYP-D3 functional and 6-311G (**d**) basis set. Adapted from [105], licensed under CC BY 4.0.

Another major line of investigation was developed by Mohammadi and collaborators, who systematically tested the binding of a broad spectrum of biologically and environmentally relevant adsorbates, including amantadine, cysteine, serine, acrolein, and hexachlorobenzene, mainly on pristine C_60_, and Si-doped and Ge-doped cages [102,103,105,106,110]. In their first contribution, the adsorption of amantadine on C_59_Si showed a dramatic strengthening relative to pristine C_60_, with adsorption energies reaching −44.8 kcal/mol and HOMO–LUMO gap reductions of ~43%, confirming the transition from weak physisorption to strong chemisorption [110]. Subsequent DFT studies on adsorption cysteine and serine on pristine C_60_, Si-doped (C_59_Si), and Ge-doped (C_59_Ge) fullerenes demonstrated that Si-doping significantly enhances the adsorption affinity of fullerene nanocages toward amino acids [105,106]. The adsorption energies for C_59_Si were notably more negative, indicating stronger binding than those for undoped C_60_. Bond index analyses, including Wiberg and Mayer indices, further confirm the greater interaction strength, revealing that Si-doped fullerenes mediate not just stronger van der Waals forces but also increased charge transfer and electronic perturbations upon adsorption. QTAIM and NCI analyses corroborate that the interactions remain non-covalent but are considerably intensified by Si substitution, suggesting that Si-doped fullerenes are more selective and sensitive nanocarriers for bioactive molecules compared to their pristine counterparts. Figure 8. displays the most stable optimized geometries for the adsorption complexes of serine on pristine, Si-doped, and Ge-doped C_60_ fullerenes, highlighting how the introduction of a silicon atom into the fullerene cage promotes a closer and more favorable association between the serine molecule and the nanocage, as evidenced by the reduced intermolecular distance and distinct coordination. This structural arrangement is consistent with the enhanced adsorption energies and increased bond indices reported for the Si-doped system.

Environmental targets such as acrolein, a toxic aldehyde, and hexachlorobenzene, a persistent pollutant, revealed a similar pattern, i.e., C_59_Si complexes stabilized with adsorption energies (E_ads_) around −47 to −50 kcal/mol, and exhibited frontier orbital localizations on the Si site, enhancing sensitivity for pollutant detection. The uniform use of DFT protocols (B3LYP-D3, wB97XD, M06-2X/6-311G(d)) combined with QTAIM, NBO, and NCI analyses allowed direct cross-comparison, consistently showing that Si doping not only strengthens adsorption but also amplifies spectroscopic and electronic signatures more effectively than pristine C_60_ or alternative cages. Altogether, these results identify Si-doped fullerenes as highly reactive and electronically responsive nanoplatforms for drug delivery and chemical sensing [102,103]. By applying a similar protocol across multiple targets, Mohammadi and collaborators provided compelling evidence for the broad applicability of Si-doping in nanomedicine and pollutant capture.

Another research strand has focused on neurotransmitters and larger biomolecules. Yadav and collaborators demonstrated that C_59_Si exhibits markedly stronger binding to histamine (≈−37.4 kcal mol^−1^) and epinephrine (≈−37.3 kcal mol^−1^) compared to pristine C_60_ (≈−1.7 kcal mol^−1^), with substantial charge transfer (up to ~0.27 e to the cage) and significant band-gap narrowing; importantly, the interactions remained thermodynamically favorable in aqueous media (ΔG < 0), confirming the potential of Si-doped fullerenes as robust biosensors [107,109]. For tyramine, Pattanaik et al. reported BSSE-corrected adsorption energies of about −25.5 kcal mol^−1^ on C_59_Si (compared to only −1.5 kcal mol^−1^ on pristine C_60_), indicating a transformation from weak physisorption to strong chemisorption. This stabilization was accompanied by HOMO–LUMO gap narrowing from 2.75 eV (pristine C_60_) to 2.07 eV on C_59_Si, enhancing conductivity and signaling the potential for electronic readout. A NBO analysis further showed a net charge transfer of 0.257 e from tyramine to the Si-doped cage, while NCI/RDG plots revealed significant noncovalent contributions (C–H⋯π, N–H⋯π) supplemented by partial covalent interactions, jointly explaining the strong adsorption and sensor suitability. In comparison, B-doping also enhanced binding relative to pristine C_60_ (E_ads_ ≈ −20.8 kcal mol^−1^) and induced gap reductions, but the electronic perturbations and charge transfer (0.174 e) were smaller than in the Si-doped case, underscoring the stronger reactivity and sensing potential of C_59_Si [108].

Expanding upon the topic of adsorption on Si-doped C_60_ to APIs (active pharmaceutical ingredients), Silva and collaborators provided a detailed comparison of carbamazepine (CBZ) adsorption on pristine and doped fullerenes, showing that C_59_Si forms a stable chemisorptive complex with E_ads_ ≈ −1.96 eV in water (BSSE-corrected), significantly stronger than the negligible binding on pristine C_60_. QTAIM analyses revealed a partial covalent Si⋯O interaction with the CBZ amide oxygen, while RDG surfaces indicated complementary van der Waals contributions. Importantly, electrostatic potential (ESP) mapping showed that C_59_Si exhibits the most positive charge accumulation on the pentagon face adjacent to Si, which orients CBZ for stronger stabilization compared to C_59_Ga, despite Ga’s higher intrinsic electrophilicity. This finding highlights that Si substitution not only lowers the HOMO–LUMO gap (~1.39 eV in water vs. 1.90 eV for pristine C_60_) but also enhances directional binding through local ESP effects, making C_59_Si a promising candidate for aqueous CBZ detection and capture [24].

Finally, for the bioactive polyphenol EGCG (epigallocatechin-3-gallate), Singh et al. conducted a systematic comparison of pristine C_60_ with heterofullerenes doped by Si, Al, Ga, B, Ge, and P. Among these, C_59_Si afforded substantial adsorption (E_ads_ ≈ −1.421 eV), markedly stronger than pristine C_60_ (−0.358 eV), and delivered the smallest band gap among all dopants (≈1.55 eV), pointing to enhanced electronic sensitivity. HOMO–LUMO plots showed clear electron density delocalization from the cage into EGCG upon binding, while QTAIM/NCI analyses identified stabilizing Si–O contacts (bond length ~1.76 Å, WBI ≈ 0.05) indicative of weakly covalent interactions. By contrast, Al-doped fullerenes exhibited the highest adsorption energy (−2.128 eV) but retained a wider band gap (1.94 eV), and B-doped systems showed shorter B–O bonds (1.60 Å), yet weaker overall adsorption (−0.80 eV). Thus, while Al maximized binding strength and B introduced strong localized interactions, Si-doping uniquely combined moderate-to-strong adsorption with the most pronounced band gap narrowing, enhancing conductivity and signal responsiveness, features highly desirable for biosensing applications. Importantly, recovery time analysis predicted that C_59_Si complexes could achieve practical desorption rates (τ ≈ 10^11^–10^5^ s depending on T), whereas Al-doped analogs were essentially irreversible under ambient conditions, underscoring the controllability of Si sites. Collectively, these results highlight Si-doped fullerenes as particularly balanced candidates, offering strong but reversible adsorption and large electronic perturbations, a triad of properties critical for biosensor and environmental detection platforms [118]. In turn, Bashiri and collaborators demonstrated that Si-doped fullerenes act as highly effective work-function type sensors for amphetamine (AA). Their DFT calculations (B3LYP-D/3-21G*, GAMESS) revealed that pristine C_60_ interacts only weakly with amphetamine (E_ads_ ≈ −0.4 kcal mol^−1^), whereas substitutional Si doping enhances adsorption energies dramatically, reaching nearly −50 kcal mol^−1^. Upon adsorption, the SiC_59_ system exhibited a substantial upward shift in the Fermi level and a marked decrease in the work function (up to 23%), indicating increased electron emission and sensor responsiveness. DOS analyses further showed that Si-doping introduces new localized states near the Fermi level, amplifying conductivity changes upon guest binding. Compared to the other dopants investigated (B, Al, Ga, Ge), SiC_59_ provided one of the strongest binding affinities and the largest electronic perturbations, thus highlighting its superior suitability for drug-sensing applications [74].

Parallel research has addressed environmental and ionic sensing, again underscoring the distinctive role of Si substitution. Amiraslanzadeh and collaborators compared several dopants for H_2_S, SO_2_, and thiophene detection. Their investigation showed that, although Al- and N-doped cages produced the strongest adsorption, SiC_59_ still improved sensitivity relative to pristine C_60_ [93]. Yousefian et al. quantified this in detail by analyzing H_2_S adsorption on pristine and doped C_60_ cages. For pristine C_60_, the interaction was essentially negligible, with an adsorption energy of only ≈−0.05 kJ mol^−1^, indicating weak physisorption and minimal perturbation of the electronic structure. Substitutional doping with silicon, however, markedly strengthened the interaction, yielding adsorption energies of ≈−10.6 kJ mol^−1^ and inducing measurable electronic changes, including a band gap reduction of ~1.1% and localized charge transfer to the dopant site. While this enhancement was noticeably weaker compared to boron doping—which produced much stronger binding, up to −32.3 kJ mol^−1^—Si-doping nonetheless transformed the C_60_ from an inert host into a responsive sensor platform [92]. In turn, Hassanpour and collaborators further demonstrated that SiC_59_ is particularly selective toward divalent cations, with adsorption energies of ≈−11.2 eV for Be^2+^ and ≈−11.0 eV for Mg^2+^ (compared to ≈−4.6 eV on pristine C_60_), while monovalent ions such as Li^+^ and Na^+^ bound much more weakly (≈−2.9 and −2.1 eV, respectively). Detailed AIM and NBO analyses revealed that divalent cation binding to the Si site exhibits partially covalent character, as reflected by higher electron density at the bond critical points relative to pristine C_60_. DOS and frontier orbital plots further confirmed that adsorption of Be^2+^ or Mg^2+^ markedly narrows the HOMO–LUMO gap (from 2.76 eV to ~1.5 eV), thereby enhancing conductivity and suggesting strong prospects for electronic sensing of alkaline-earth cations [90]. Overall, these studies confirm that, while Si doping is not the strongest dopant across all analytes, it consistently converts weak physisorption into measurable chemisorption, imparting predictable and tunable electronic responses that are particularly advantageous for selective environmental sensing.

Regarding the research on Si-doped fullerenes in the field of energy storage and catalysis, Ganji et al. showed that each substitutional Si site can adsorb up to six H_2_ molecules with the average binding energies of 0.45 eV for the first layer and 0.11 eV for the second, enabling six dopants to host as many as 36 H_2_ (7.5 wt%), surpassing the DOE gravimetric storage target. This capacity compared favorably to S- and P-doped cages, which stabilized only five and two H_2_ per dopant, respectively [58]. In turn, Pei and collaborators systematically evaluated Li^+^ transport and found that, while pristine C_60_ exhibits a migration barrier of 0.19 eV, Si substitution lowers it dramatically to 0.038 eV, enhancing ionic mobility and identifying C_59_Si as a promising fast-charging electrode material. B doping increased the barrier to 0.37 eV, highlighting silicon’s distinctive facilitation of Li^+^ diffusion [91].

In catalysis, Deddouche and Chemouri compared CO oxidation performance on B-, Si-, P- and S-doped C_60_ cages. The results showed a clear hierarchy: B- and P-doping provided only moderate stabilization, with CO adsorption energies of about −1.25 and −1.64 eV, respectively, while S-doping was even weaker (−0.98 eV), offering little improvement over pristine C_60_. In sharp contrast, SiC_59_ exhibited the strongest interaction with CO (−2.84 eV) and with O_2_ (−3.08 eV), accompanied by the highest electrophilicity index (ω ≈ 5.36 eV) and the narrowest HOMO–LUMO energy gap (Eg ≈ 1.44 eV). These features collectively enabled more efficient O–O bond activation and stabilization of the CO–O_2_ coadsorbed complex, thereby lowering the barriers of the Langmuir–Hinshelwood pathway. By comparison, the weaker adsorption and lower polarity of B-, P-, and S-doped cages limited their catalytic utility, underscoring that substitutional Si provides the optimal balance of binding strength and electronic activation for metal-free CO oxidation catalysts [94].

**Figure 9:**
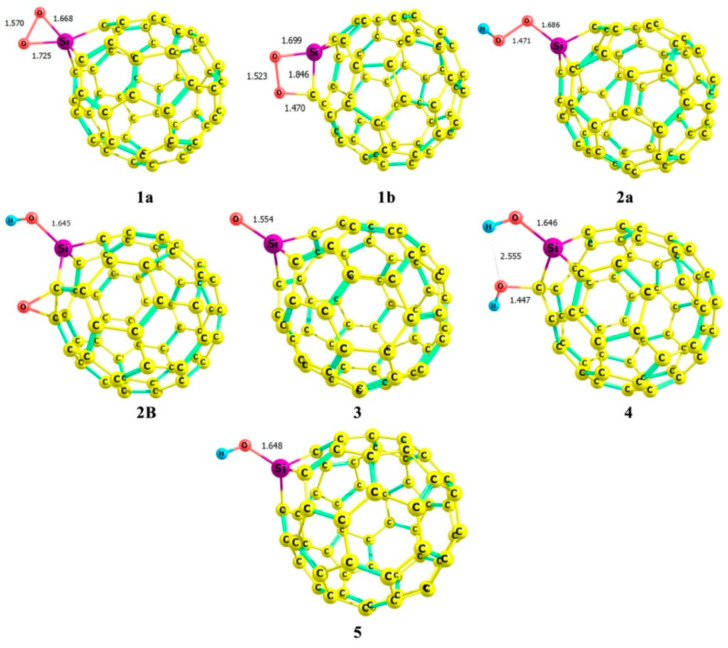
Structures of key intermediates involved in ORR catalytic cycle on Si-doped fullerene C_59_Si: “atop” (**1a**) and “bridge” (**1b**) O_2*_ C_59_Si, HOO_*_C_59_Si (**2a**), HOO_*_C_59_Si (**2b**), O_*_C_59_Si (**3**), HO_*_HO_*_ C_59_Si (**4**), and HO_*_ C_59_Si (**5**), calculated at the B3LYP/6-311+G(d) level of theory. Used with permission from [95]; permission conveyed through Copyright Clearance Center, Inc.

In turn, Vashchenko et al. traced the oxygen reduction reaction (ORR) mechanism on C_59_Si. The authors showed that oxygen adsorption on C_59_Si preferentially forms a four-membered siladioxetane “bridge” intermediate (E_ads_ ≈ −3.55 eV), which is ~0.8 eV more stable than the siladioxirane “atop” configuration, directly contradicting earlier assignments. Sequential proton–electron transfers yield highly stabilized species such as HOO C_59_Si (ΔG = −3.57 eV) and O C_59_Si (E_ads_ ≈ −7.18 eV), reflecting silicon’s strong oxygen binding propensity and its effectiveness in promoting O–O bond dissociation. Free energy profiles confirm that the 4e^−^ pathway to H_2_O is favored over the 2e^−^ peroxide route in both acidic and alkaline environments, though the catalytic cycle terminates at different intermediates, depending on electrode potential (Figure 9). Notably, Si doping narrows the HOMO–LUMO gap after O_2_ adsorption and enables a barrierless exchange in which O_2_ displaces H_2_O, thereby sustaining catalytic activity. These findings established SiC_59_ as a viable, metal-free ORR catalyst with mechanistic features distinct from B- or N-doped analogs [95].

Collectively, these studies highlight that silicon substitution not only enhances hydrogen storage and lithium-ion kinetics but also endows fullerenes with catalytic functionality for both oxidation reactions and energy conversion, underlining their potential as metal-free multifunctional nanomaterials. Table 4. provides a summary of the computational protocols applied to the above-described studies, specifying the semiempirical methods, DFT functionals, and basis sets used in the respective research on exohedral complexes of substitutional Si-doped C_60_ fullerenes.

**Table 4:** Selected articles on application of theoretical calculations on exohedral complexes of substitutional Si-doped C_60_ fullerenes.

Exohedral Complexes of Substitutional Si-Doped C_60_ Fullerenes							
Si-Doped Fullerene Derivative (Host)	Adsorbed Guest	Type of Calculation	Calculation Method		Computational Software	Aim of Study	(Ref.)
			Semiempirical	DFT (Functional and Basis Set)			
C_60−n_Si_n_ (n = 1, 2, 4, 6)	mH_2_ (m = 1–6, 12, 24, 36)	GO, ESPA, E_ads_	SCC-DFTB	LDA/DPZ	SIESTA code DFTB+ code	Evaluation of H_2_ adsorption capacity and binding energy on Si-doped C_60_ via exohedral interaction (also S-doped and P-doped C_60_)	[58]
C_59_Si	Li^+^	GO, ESPA, CINEB, E_ads_	-	PBE-D3	VASP	Investigation of Si-doping effects on structural stability, electronic properties, and lithium-ion adsorption/migration in C_60_ (also C_59_B, C_59_P, C_59_S, C_59_N, and pristine C_60_)	[91]
C_59_Si	SO_2_, H_2_S, thiophene	GO, ESPA, PCM	-	ωB97XD/6-31+G(d)	Gaussian 09	Evaluation of adsorption capacity of C_59_Si toward SO_2_, H_2_S and thiophene in gas and aqueous phase (also C_59_B, C_59_P, C_59_S, C_59_N, and C_59_Al)	[93]
C_59_Si	CO, O_2_	GO ESPA E_ads_ (with BSSE), PCM	-	B3LYP-gCP-D3/6-31G(d)	Gaussian 09	Assessment of O_2_ and CO co-adsorption and catalytic oxidation activity of C_59_Si (also C_59_B, C_59_P, C_59_S, and pristine C_60_)	[94]
C_59_Si	O_2_, HOO·, HO·O·, O·, HO·HO·, HO·	GO, ESPA, E_ads_		B3LYP/6-311+G(d), B3LYP/6-311G(d,p), wB97XD/6-311G(d,p)	Gaussian09	Evaluation of adsorption and activation of oxygen reduction reaction intermediates on Si-doped fullerene catalyst C_59_Si (in comparison to C_60_)	[95]
C_59_Si	amphetamine	GO, ESPA, E_ads_ (with BSSE), NICS	-	B3LYP-D/3-21G(d), B3LYP-D/6-31G, B3LYP-D/6-311++G(d,p), GIAO	GAMESS	Investigation of the adsorption process and aromaticity response of Si-doped C_60_ as a molecular sensor for amphetamine (in comparison to C_59_B, C_59_Ga, C_59_Ge, C_59_Al, and pristine C_60_)	[74]
C_59_Si	5-fluorouracil	GO, ESPA, E_ads_ (with BSSE), PCM	-	B3LYP/6-31G(d), ωB97x-D/6-31G(d), M06-2X/6-31G(d)	GAMESS	Evaluation of the adsorption affinity and electronic effects of 5-fluorouracil on Si-doped C_60_ (in comparison to C_59_B, C_59_Al, and pristine C_60_)	[75]
C_59_Si	6-chloro-3-hydroxy-2-pyrazinecarboxamide	GO, ESPA, E_ads,_ PCM	-	B3LYP/6-31G(d), M06-2X/6-31G(d)	Gaussian 09	Evaluation of adsorption energy and electronic properties of Si-doped C_60_ interacting with pyrazinamide derivative (in comparison to C_59_Al, and pristine C_60_)	[121]
C_59_Si	amantadine	GO, ESPA, E_ads_ (with BSSE), IR, NMR, PCM	-	B3LYP/6-31G(d), B3LYP/cc-pVDZ	Gaussian 09	Investigation of noncovalent interactions between Si-doped C_60_ and amantadine in gas and aqueous phases (in comparison to C_59_B, C_59_Ga, C_59_Ge, C_59_Al, and pristine C_60_)	[62]
C_59_Si	favipiravir	GO, ESPA, BSSE, E_ads,_ PCM	-	M06-2X/6-31G(d)	Gaussian 09	Investigation of the adsorption mechanism, and the structural and electronic properties of favipiravir on Si-doped C_60_ and pristine C_60_ in gas and aqueous phases for potential drug delivery applications	[63]
C_59_Si, C_58_Si_2_	2,4,6-trinitrotoluene (TNT)	GO, ESPA, E_ads_	-	B3LYP/6-31G(d)	Gaussian 09	Assessment of the adsorption mechanism, stability, reactivity and sensing performance of single- and double-Si-doped C_60_ toward explosive substance TNT in gas phase	[96]
C_59_Si	β-propiolactone	GO, ESPA, E_ads_ (with BSSE), PCM	-	B3LYP/6-31G(d)	Gaussian 09	Investigation of the adsorption properties, electronic structure, and solvent effects on β-propiolactone binding to Si-doped C_60_ (also C_59_Al) for potential sensing applications	[99]
C_59_Si	paracetamol	GO, ESPA, BSSE, E_ads_ (with BSSE), PCM	-	B3LYP/6-31G(d)	Gaussian 16	Assessment of the adsorption strength, structural and electronic properties of paracetamol binding to Si-doped C_60_ (also other C_60_ derivatives) for potential drug delivery and sensing applications	[112]
C_59_Si	amantadine	GO, ESPA, E_ads_	-	HSEH1PBE/6-311G(d), B3LYP-D3/6-311G(d), wB97XD/6-311G(d), M062X/6-311G(d)	Gaussian 16	Assessment of the adsorption strength and electronic reactivity descriptors for amantadine on Si-doped C_60_ (in comparison to C_59_B, C_59_Ga, C_59_Ge, C_59_Al, C_59_P, C_59_As, C_59_N, and pristine C_60_)	[110]
C_59_Si	4-phenylpyridine (4-PHPY)	GO, ESPA, E_ads_	-	M062X/6-31G(d), B3LYP/6-31G(d)	Gaussian 09	Evaluation of the adsorption strength and electronic properties of 4-PHPY on Si-doped C_60_ (in comparison to C_59_B)	[98]
C_59_Si	piperazine-2,3,5,6-tetraone (PPTO)	GO, ESPA, E_ads_ (with BSSE), PCM, NMR	-	B3LYP/6-31G(d)	Gaussian 09	Evaluation of the adsorption strength and electronic reactivity of Si-doped C_60_ as drug carrier or sensor for PPTO (in comparison to C_59_Al)	[100]
C_59_Si	1-(3-trifluoromethylphenyl)piperazine (TFMPP)	GO, ESPA, E_ads_ (with BSSE),	-	B3LYP/cc-pVDZ	Gaussian 09	Assessment of binding affinity and sensing ability of Si-doped C_60_ (in comparison to C_59_Al) for an ecstasy analog (TFMPP) with CNS activity.	[101]
C_59_Si	phenylalanine	GO, ESPA, E_ads_, IR	-	B3LYP/6-31G(d), M062X/6-311G(d)	Gaussian 09	Investigation of the structural stability, charge distribution and adsorption behavior of phenylalanine on Si-doped C_60_ in gas and aqueous phase (in comparison to C_59_Al and pristine C_60_)	[104]
C_59_Si	metronidazole	GO, ESPA, E_ads_	-	B3LYP/6-31G(d,p)	Gaussian 09	Investigation of the electronic structure, adsorption energy and quantum reactivity descriptors of metronidazole adsorbed on Si-doped C_60_ (in comparison to C_59_B, C_59_Al, and pristine C_60_)	[114]
C_59_Si	ornidazole	GO, ESPA, E_ads_	-	B3LYP/6-31G(d)	Gaussian 09	Investigation of the adsorption energy, molecular electrostatic potential and frontier orbital properties of ornidazole on Si-doped C_60_ (in comparison to C_59_B, C_59_Al and pristine C_60_)	[25]
C_59_Si	molnupiravir	GO, ESPA, E_ads_, PCM, IR	-	B3LYP/cc-pVDZ	Gaussian 09	Investigation of the quantum reactivity descriptors, adsorption mechanism and intermolecular interactions between Si-doped C_60_ and molnupiravir	[111]
C_59_Si	carbamazepine	GO, ESPA, E_ads_ (with BSSE), PCM	-	ωB97XD/6-31G(d), M06L/6-31G(d)	Gaussian 09	Investigation of the interaction energy, solvent effects, and electronic properties of carbamazepine adsorbed on Si-doped C_60_ (in comparison to C_59_B, C_59_Ga, C_59_Ge, C_59_Al, C_59_P, C_59_N and pristine C_60_)	[24]
C_59_Si	nimesulide, diclofenac, mefenamic acid	GO, ESPA, E_ads_ (with BSSE), PCM, SMD, IR	-	B3LYP/6-31G(d)	Gaussian 09	Investigation of the solvent effects, electronic structure, and nonlinear optical properties of Si-doped C_60_ interacting with NSAID drugs (in comparison to pristine C_60_)	[116]
C_59_Si	histamine	GO, ESPA, E_ads_ (with BSSE), PCM	-	B3LYP-D3/6-31G(d,p)	Gaussian 16	Investigation of the adsorption behavior, electronic reactivity descriptors and solvent effects in histamine detection by Si-doped C_60_ (in comparison to C_59_B and pristine C_60_)	[107]
C_59_Si	tyramine	GO, ESPA, E_ads_ (with BSSE), MD	-	B3LYP/6-31G(d,p)	Gaussian 16	Investigation of the non-covalent interactions, adsorption stability and electronic reactivity of Si-doped C_60_ toward tyramine (in comparison to C_59_B and pristine C_60_)	[108]
C_59_Si	hydroxyurea, paracetamol	GO, ESPA, E_ads_	-	PBE	PHASE/0 code	Investigation of the adsorption energy, charge transfer and electronic properties of pharmacologically active compounds paracetamol and hydroxyurea adsorbed on Si-substituted C_60_	[113]
C_59_Si	methadone	GO, ESPA, E_ads_ (with BSSE), TDDFT	-	M062X/6-31G(d), B3LYP/6-31G(d)	GAMESS	Investigation of the structural, spectroscopic and electronic response of Si-doped C_60_ to methadone adsorption using TDDFT and quantum reactivity descriptors (in comparison to C_59_B, C_59_Ge, and pristine C_60_)	[14]
C_59_Si	epigallocatechin-3-gallate (EGCG)	GO, ESPA, E_ads_ (with BSSE)		B3LYP-D3/6-31G(d)	Gaussian 16,	Analysis of the non-covalent interactions and electronic reactivity in EGCG adsorption on Si-substituted C_60_ (in comparison to C_59_B, C_59_Ga, C_59_Ge, C_59_Al, C_59_P and pristine C_60_)	[118]
C_60−n_Si_n_ (n = 2, 4, 6, 8)	1,3-butadiene	GO, ESPA, E_ads_ (with BSSE), NICS	-	M06-2X/6–311+G(d)	GAMESS	Investigation of the aromaticity and Diels–Alder reactivity of Si-substituted C_60_ with increasing silicon content	[88]
C_60−n_Si_n_ (n = 1–4, 6), C_60_Si_m_ (m = 1–6)	Si_m_ (m = 1–6)	GO, ESPA, MD	DFTB-based TBMD	-	DFTB code	Investigation of the fragmentation dynamics of substitutional and adsorbed Si-doped C_60_ fullerenes	[60]
C_60−n_Si_n_ (n = 1), C_60_Si_m_ (m = 1–2)	Si_m_ (m = 1, 2)	GO, ESPA, MD	-	PW91	“Dacapo” code	Investigation of the thermal stability, structural and electronic properties of substitutional vs. exohedral doping behavior of Si-substituted C_60_	[45]
C_60−n_Si_n_ (n = 1–12) C_60_Si_m_ (m = 1–15)	Si_m_ (m = 1–15)	GO, ESPA, MD	DFTB-based TBMD	-	DFTB code	Investigation of the thermal behavior and fragmentation mechanisms of Si-doped C_60_ fullerenes, including substitutional derivatives (C_60−n_Si_n_) and exohedral adducts (C_60_Si_m_)	[61]
C_59_Si	Si_m_ (m = 10, 15, 20)	GO, ESPA, E_ads_	-	PW91, PAW	VASP	Investigation of the structural and electronic properties of nanostructures C_59_Si–Si_m_ (m = 10, 15, 20)	[89]
C_59_Si	phenylpropanolamine	GO, ESPA, E_ads_ (with BSSE), NICS	-	B3LYP-D/3-21G(d), GIAO	GAMESS	Investigation of the structural, electronic, adsorption and aromatic properties of C_59_Si with a pharmacologically active compound (in comparison to C_59_Al and pristine C_60_)	[120]
C_59_Si	H_2_S	GO, ESPA, E_ads_	-	B3LYP/3-21G(d)	Gaussian 09	Investigation of the structural, electronic and adsorption properties of C_59_Si for H_2_S gas sensing (in comparison to C_59_B, C_59_N, C_59_S, C_59_P and pristine C_60_)	[92]
C_59_Si	valproic acid	GO, ESPA, E_ads_ (with BSSE), PCM, IR, NMR	-	B3LYP/cc-pVDZ	Gaussian 09	Investigation of the interaction mechanism and stability of valproic acid adsorbed on Si-doped C_60_ (in comparison to C_59_B, and C_59_Al)	[115]
C_58_Si_2_	salicylic acid, flurbiprofen	GO, ESPA, E_ads_ (with BSSE), PCM, IR	-	B3LYP/6-31G(d)	Gaussian 09	Assessment of the structural, electronic and adsorption properties of C_58_Si_2_ as a nanocarrier for two NSAIDs	[119]
C_59_Si	hydroquinone	GO, ESPA, E_ads_ (with BSSE), PCM	-	B3LYP/6-31G(d), M06-2X/cc-pVDZ	Gaussian 09	Investigation of the adsorption mechanism and electronic properties of hydroquinone on Si-doped C_60_ (in comparison to C_59_B)	[97]
C_59_Si	Li^+^, Na^+^, K^+^, Be^2+^, Mg^2+^, Ca^2+^	GO, ESPA, E_ads_ (with BSSE)	-	B3LYP/6-31G(d)	GAMESS	Investigation of the structural, electronic, and adsorption properties of alkali and alkaline earth cations on Si-doped C_60_ nanocage for potential sensor applications (compared with pristine C_60_)	[90]
C_57_SiAlB	ifosfamide	GO, ESPA, E_ads_ (with BSSE), PCM, IR	-	B3LYP/6-31G(d), wB97XD/6-31G(d)	Gaussian 09	Investigation of the structural, electronic, adsorption, and vibrational properties of tri-doped C_60_ interacting with ifosfamide for potential drug delivery applications	[117]
C_59_Si	epinephrine	GO, ESPA, E_ads_, PCM	-	B3LYP/6-31G(d,p)	Gaussian 16	Evaluation of the adsorption behavior, structural, electronic, and energetic properties of Si-doped C_60_ interacting with epinephrine (compared with C_59_B and pristine C_60_)	[109]
C_59_Si	serine	GO, ESPA, E_ads_	PM6	PBE0/6-311G(d), B3LYP-D3/6-311G(d), ωB97XD/6-311G(d), M06-2X/6-311G(d)	Gaussian 16	Assessment of the intermolecular interactions, structural, electronic, and adsorption properties of Si-doped C_60_ with serine (compared with C_59_Ge and pristine C_60_)	[105]
C_59_Si	cysteine	GO, ESPA, E_ads_	PM6	PBE0/6-311G(d), B3LYP-D3/6-311G(d), ωB97XD/6-311G(d), M06-2X/6-311G(d)	Gaussian 16	Investigation of the intermolecular interactions, structural, electronic, and adsorption properties of Si-doped C_60_ with cysteine (compared with C_59_Ge and pristine C_60_)	[106]
C_59_Si	acrolein	GO, ESPA, E_ads_	PM6	PBE0/6-311G(d), B3LYP-D3/6-311G(d), ωB97XD/6-311G(d), M06-2X/6-311G(d)	Gaussian 16	Investigation of the intermolecular interactions, structural, electronic, and adsorption properties of Si-doped C_60_ with acrolein (compared with C_59_Ge and pristine C_60_)	[102]
C_59_Si	Hexachlorobenzene (HCB)	GO, ESPA, E_ads_	PM6	PBE0/6-311G(d), B3LYP-D3/6-311G(d), ωB97XD/6-311G(d), M06-2X/6-311G(d)	Gaussian 16	Investigation of the adsorption mechanism, structural, electronic, and reactivity properties of Si-doped C_60_ with HCB (compared with C_59_Ge and pristine C_60_)	[103]
C_60−n_Si_n_ (n = 30), C_60_Si_m_ (m = 1–30)	Si_m_ (m = 1–30)	GO, ESPA, FTMD	SCC-DFTB	LSDA/6-31G(d), PBE/6-31G(d), B3LYP/6-31G(d)	Gaussian 03, “Dylax” DFTB code	Investigation of the thermal stability and formation mechanisms of substitutional and exohedral Si-doped C_60_	[59]

SCC DFTB—Self-Consistent Charge Density Functional Tight Binding, CINEB—Climbing Image Nudged Elastic Band, PCM—Polarizable Continuum Model, SMD—Solvation Model based on Density, RDG—Reduced Density Gradient Analysis, DFTB—Density Functional Tight Binding, NICS—Nucleus-Independent Chemical Shift, BSSE—Basis Set Superposition Error, CP—Counterpoise method.

In summary, computational studies have revealed a remarkably broad functional spectrum for Si-doped fullerenes—from nanocarriers and biosensors to catalysts and energy storage media. The diversity of research aims reflects the dual role of Si substitution: locally distorting and polarizing the cage while simultaneously lowering electronic gaps and introducing reactive centers. This combination underpins both their enhanced binding capacity and their unique electronic signatures, firmly establishing silicon-doped fullerenes as versatile and tunable nanostructures at the interface of materials science, chemistry, and biomedicine.

### 3.3. Endohedral Complexes of Substitutional Si-Doped C_60_ Fullerenes

Endohedral complexes of Si-doped C_60_ involve the encapsulation of atoms or small molecules within the silicon-substituted fullerene cage. Such systems offer a unique platform to probe host–guest interactions under confinement and to evaluate how substitutional silicon atoms influence the electronic and structural stability of the encapsulated complexes. Computational studies have shown that Si doping can enhance cage reactivity, alter charge transfer patterns, and modulate the energetics of endohedral binding, thereby expanding the potential of these heterofullerenes for applications in hydrogen storage, nano-containers, and molecular electronics.

To the best of our knowledge, only two theoretical publications have so far addressed endohedral complexes of Si-doped C_60_, both focusing on the C_59_Si cage with encapsulated hydrogen species. Ganji and collaborators conducted a comprehensive theoretical investigation into the formation and stability of endohedral hydrogen complexes within B-, N-, and Si-doped C_60_ fullerenes, with particular emphasis on the Si-substituted system (C_59_Si). The research examined the encapsulation of up to four molecular hydrogen (H_2_) molecules inside the fullerene cages, focusing on their geometric structure, electronic interactions, and energetics. The results showed that, while up to two H_2_ molecules form stable endohedral complexes with all cages, the 2H_2_@C_59_Si system was the most stable (ΔE ≈ −0.53 eV). Interestingly, the 3H_2_@C_59_Si complex was also stable (ΔE ≈ −0.23 eV), in contrast to the nearly metastable 3H_2_@C_60_, highlighting the role of silicon in enhancing storage capacity. Mulliken analysis indicated minor charge transfer (~0.04–0.10 eV) from the cage to the confined hydrogens. Overall, Si-doping uniquely extended the stability limit of hydrogen encapsulation, suggesting its potential for hydrogen storage materials. All the abovementioned calculations were performed using the density functional theory method and the SIESTA package, employing the generalized gradient approximation (GGA) with the Perdew-Burke-Ernzerhof (PBE) functional, Troullier-Martins norm-conserving pseudopotentials, and a double-zeta plus polarization (DZP) atomic orbital basis set [58].

In turn, Javan and collaborators performed the study of atomic hydrogen encapsulation in a series of heterofullerene nanocages (including B-, Si-, P-, O-, and S-doped C_60_) with a specific focus on the properties of single hydrogen atoms trapped in the C_59_Si cage. Their calculations centered on optimizing the molecular structures, assessing electronic configurations (such as the HOMO–LUMO gap and density of states), and determining the binding energies of the encapsulated species. Computations were carried out using density functional theory (DFT) in the OpenMX code, employing linear combination of pseudo-atomic orbitals (LCPAO) and norm-conserving pseudopotentials with either LDA or GGA functionals. The primary exchange-correlation treatment used the GGA/PBE functional and Troullier-Martins pseudopotentials, with major structural and energetic results obtained with GGA/PBE. Based on the calculation results, the authors found that hydrogen resides stably at the center of all cages, but binding energies follow the hierarchy H@C_59_O < H@C_59_Si < H@C_60_ < H@C_59_B < H@C_59_S < H@C_59_P, with H@C_59_Si giving Eb ≈ −0.22 eV (≈−0.1 eV after BSSE correction). Si substitution caused notable cage distortions (C–Si bond lengths 1.775–1.827 Å) but maintained stable encapsulation, albeit weaker than B or P. Electronic structure analysis showed altered HOMO/LUMO distributions and reduced symmetry relative to C_60_. These findings suggested that, while Si is not the strongest stabilizer, it offers a balance between structural distortion and hydrogen retention, making H@C_59_Si relevant for endohedral storage and quantum applications [122].

Both studies utilized closely related computational frameworks—primarily DFT with GGA/PBE functionals and norm-conserving pseudopotentials—albeit targeted at different encapsulated hydrogen species: Ganji et al. focused on molecular H_2_, whereas Javan and collaborators analyzed atomic H. In both cases, the Si-doped C_60_ cage (C_59_Si) was found to accommodate a limited number of hydrogen species with weak physisorption and minimal structural distortion of either the guest or the host, and only marginally negative formation energies for increased loading. The studies confirmed that encapsulation of hydrogen—whether atomic or molecular—in C_59_Si leads to weak binding with marginal thermal stability, insufficient for practical hydrogen storage objectives but providing a unique test-bed for the study of host-guest and dopant-guest interactions in fullerene nanoscience [58,122].

Table 5 provides a summary of the computational protocols applied, specifying the semiempirical methods, DFT functionals, and basis sets used in the respective studies on endohedral complexes of substitutional Si-doped C_60_ fullerenes.

## 4. Conclusions

In summary, silicon-doped fullerenes present unique structural and electronic behaviors arising from the subtle interplay between silicon’s preference for sp^3^ hybridization and carbon’s versatile sp^2^ bonding networks. Experiments demonstrate the viability of substituting multiple Si atoms into fullerene cages without destabilizing their spherical geometry, yet also reveal pronounced bond length distortions, altered HOMO–LUMO gaps, and localized electronic states on Si atoms. The complex rearrangements, charge transfer phenomena, and chemical reactivity at Si sites challenge existing computational approaches, necessitating advanced first-principles and electronic structure methods that can reliably capture the peculiarities of Si-C bonding and cage deformation. Expanding and refining these theoretical tools will accelerate our understanding of stability, electronic properties, and functionalization potential, and enable the predictive design of novel Si-containing carbon nanomaterials.

## Figures and Tables

**Table 1:** Representative studies of Si-doped fullerenes: synthesis methods, analytical techniques, proposed products, and limitations.

Study	Synthesis Method	Analytical Techniques	Proposed Products/Findings	Limitations
Kimura et al. (1996) [46]	Laser vaporization of Si-doped graphite	TOF-MS	SiC_n_ (n ≈ 56–61) clusters in fullerene-size window; heterofullerene-like distributions	Cannot distinguish substitutional vs. exohedral MS fragmentation (C_2_ loss); complicates interpretation
Fye and Jarrold (1997) [48]	Laser vaporization; drift-tube ion mobility with MS	Ion mobility + MS, fragmentation, water-adduct reactivity	C_2n−1_Si^+^ consistent with substitutional doping; C_2n_Si^+^ consistent with exohedral Si	Gas-phase only; no bulk isolation
Cao et al. (1997) [47]	Arc evaporation of SiC-loaded electrodes; extraction in CS_2_	MS, HPLC	Bulk Si-containing fullerene fractions obtained	No unambiguous assignment between substitutional and exohedral structures
Ray et al. (1998) [23]	Laser vaporization of Si:C mixed targets (carbon-rich; clusters formed as cations)	Reflectron TOF-MS (abundance patterns) and excimer-laser photofragmentation of size-selected clusters	C_2n−q_Si_q_ (q = 1, 2) mirror pure fullerene stability pattern; first neutral losses are SiC and Si_2_ from Si-doped parents, then “fullerene-like” C_2_ losses; spectra support cagelike geometry with neighboring Si atoms	Gas-phase only; mass coincidences prevent unique assignment for >2 Si; no bulk isolation/crystallography.
Pellarin et al. (1999) [49]	Laser vaporization from Si_x_C_1−x_ mixed targets (x = 0–50%); both stoichiometric SiC and carbon-rich; photoionization/photofragmentation sequence	High-fluence photoionization MS of neutrals; photofragmentation MS of size-selected cations	Laser annealing yields stable Si-doped fullerenes; early losses of Si_2_C/Si_3_C; patterns indicate cagelike substitution with up to ~12 Si atoms tolerated	Inferences based on MS patterns; isomass overlaps complicate assignments at higher number of Si atoms; no structural (solid-state) confirmation.
Billas et al. (1999) [52]	Inert-gas condensation: mixing preformed C_60_ vapor with laser-vaporized Si in He; subsequent photoionization/photofragmentation	TOF-MS (photoionization), photofragmentation of C_60_Si_x_ parents; plus ab initio DFT support	Transformation from exohedral C_60_Si_x_ clusters to in-cage (substitutional) C_59_Si/C_58_Si_2_ upon photofragmentation; evidence for ≥3 Si substitution deduced from fragment patterns despite mass coincidence	Assignment beyond two Si limited by mass coincidence; conclusions rely on fragmentation diagnostics; no bulk isolation/crystal structures.
Ohara et al. (2002) [53]	Two-rod, two-laser vaporization (C_60_ and Si/Ge) in He carrier gas	TOF-MS of anions/cations; anion photoelectron spectroscopy (PES) with magnetic-bottle detector	Exohedral C_60_Si_n_/C_60_Ge_m_ formed; size distributions limited to n ≤ 4 (m ≤ 3); PES shows that added Si/Ge largely cluster on the surface; electronic signatures for n = 3–4 resemble C_60_^−^ (charge remains on cage)	Gas-phase clusters only; no evidence for substitutional incorporation; hot-source conditions limit survival of larger n; no extractable bulk material.
Bulina et al. (2007) [54]	Atmospheric-pressure arc in carbon–He plasma with injected Si powder (AC 44 kHz); soot collected	Workup: benzene extraction (pure fullerenes), then pyridine extraction (derivatives); MS (laser desorption TOF); XRD; emission spectral analysis	MS of pyridine extract shows peaks assigned to C_52_Si_8_^+^ and C_62_Si_8_^+^ (content ≤ 1%); C_52_Si_8_/C_62_Si_8_ ≈ 7/3 (analogous to C_60_/C_70_ ratio); discussion consistent with closed cages and adjacent Si atoms	Very low abundance; assignment based solely on MS in extract; no high-resolution structural proof or IMS; alternative interpretations (fragments/adducts) cannot be fully excluded.

**Table 2:** Bond lengths (Å) of Si-doped C_60_ isomers (hh, hp, and pp stand for hexagon–hexagon, hexagon–pentagon, and pentagon–pentagon bonds) [66].

Species and Isomer	C–C (Å)	C–Si (Å)	Si–Si (Å)
C_59_Si	1.40–1.43 hh; 1.46–1.51 hp	1.848 hh; 1.900 hp	…
C_58_Si_2_, 5	1.40–1.45 hh; 1.47–1.50 hp	1.862 hh; 1.88–1.92 hp	…
C_58_Si_2_, 2	1.40–1.44 hh; 1.46–1.50 hp	1.847 hh; 1.916 hp	2.303 hp
C_58_Si_2_, 9	1.40–1.43 hh; 1.46–1.51 hp	1.848 hh; 1.900 hp	…
C_54_Si_6_, A	1.40–1.43 hh; 1.45–1.50 hp	1.872 hh; 1.928 hp	2.661 hh; 2.411 hp
C_54_Si_6_, B	1.41–1.42 hh; 1.46–1.53 hp	1.90–1.98 hp	2.360 hh; 2.372 hp
C_48_Si_12_	1.41–1.44 hh; 1.46–1.48 hp	1.83–1.85 hh; 1.82–1.84 hp	2.425 hh; 2.32–2.48 hp

**Table 3:** Selected articles on application of theoretical calculations on substitutional Si-doped C_60_ fullerenes.

Substitutional Si-Doped C_60_ Fullerenes						
Si-Doped C_60_ Derivative	Type of Calculation	Calculation Method		Computational Software	Aim of Study	First Author/Publication Year DOI (Ref.)
		Semiempirical	DFT (Functional and Basis Set)			
C_59_Si	GO, ESPA, LR-TDDFT, QR-TDDFT, PCM	-	BLYP/3-21+G(d), B3LYP/3-21+G(d), B3LYP/6-31G(d), B3LYP/cc-pVDZ, B3LYP/aug-cc-pVDZ, CAMB3LYP/6-31+G(d), LDA/3-21+G(d), LDA/6-31+G(d), BP86/3-21+G(d), BP86/6-31+G(d), PBE/6-31+G(d)	Gaussian 03, DALTON2013	Investigation of OPA/TPA spectra of Si-monosubstituted fullerenes (C_n−1_Si for n = 20, 30, 40, 50, and 60) in gas/solution to assess substitution and solvent influences	[64]
C_59_Si	GO, ESPA	PM3	-	Gaussian 98	Investigation of structural deformation and electronic localization in Si-substituted C_60_	[55]
C_59_Si	GO, ESPA, TDDFT, IR, NICS	-	B3LYP/6-31G(d), GIAO	Gaussian 09	Investigation of structural, electronic, vibrational, dielectric and aromatic property changes in C_59_Si relative to pristine C_60_ and C_60_ with other dopants (B, N, Al, As, P, Ga, Ge)	[65]
C_59_Si	GO, ESPA IR, SCRF,	-	B3LYP/aug-cc-pVDZ	Gaussian 98	Investigation of structural, energetic and vibrational differences in Si-substituted C_59_Si compared to C_60_ and C_59_N	[68]
C_59_Si, C_59_Si^6−^	GO, ESPA, NICS	-	B3LYP/6-31G(d), SCF/3-21G, GIAO	Gaussian 98	Investigation of the geometry, electronic structure, and magnetic properties of Si-doped fullerene C_60_ and C_60_^6−^ as isoelectronic analogs of C_60_ and C_60_^6−^	[72]
C_60−n_Si_n_ (n = 1, 2)	CPMD	-	BLYP	CPMD code	Investigation of structural deformation, charge localization and bonding patterns in C_59_Si and C_58_Si_2_ relative to C_60_	[52]
C_60−n_Si_n_ (n = 1, 2, 3, 6, 12)	GO, ESPA	-	LDA(PZ)/TZP (TM)	SIESTA code	Investigation of stability, structural preference and thermal fragmentation behavior of Si-substituted C_60_ isomers	[69]
C_60−n_Si_n_ (n = 1, 2, 6, 12)	GO, ESPA, TDDFT, CPMD	-	BLYP, PBE, LDA	SIESTA code, OCTOPUS 2.0.1., CPMD code	Investigation of photoabsorption spectra of Si-substituted C_60_ (up to C_48_Si_12_) to assess spectral changes upon increasing silicon content	[66]
C_60−n_Si_n_ (n = 1, 2, 6, 12, 20, 24, 30)	GO, NICS, NMR	-	B3LYP/6-311G(d), GIAO	Gaussian 98	Investigation of electronic structure and ^13^C NMR shielding parameters (NICS) in Si-substituted C_60_ to assess the impact of silicon doping on aromaticity	[67]
C_60−n_Si_n_ (n = 5, 6, 10, 12, 14, 18, 22, 24, 26, 30)	GO, ESPA, CMCS, GA (CBG model)	MNDO	B3LYP/3-21G, B3LYP/6-31G(d), PBE/DNP	Gaussian 03, DMol3	Investigation of structural stability and Si-substitution patterns in C_60_	[56]
C_60−n_Si_n_ (n = 20, 24, 30)	GO, ESPA, CPMD	-	BLYP, TM-NCPP	CPMD code	Investigation of structural stability and charge transfer in highly Si-substituted C_60_	[77]
C_60−n_Si_n_ (n = 20, 24, 30)	GO, ESPA, FPMD	-	BLYP, TM-NCPP	CPMD code	Investigation of thermal instability threshold and fragmentation mechanisms in highly Si-doped C_60_	[76]
C_30_Si_30_	GO, ESPA, CPMD	-	BLYP	CPMD code	Investigation of effects of ±1e charge on structure and electronic properties of Si-substituted C_60_	[78]
C_30_Si_30_	GO, ESPA, FPMD	-	PBE-PP, BLYP-PP, BLYP/6-311G, BLYP/6-311G(d), B3LYP/6-311G, B3LYP/6-311G(d), BLYP/TZV, BLYP/TZVP	CPMD code, Gaussian 09	Investigation of topological conditions underlying the thermal stability of C_30_Si_30_	[70]
C_60−n_Si_n_ (n = 1–4)	GO, ESPA	MNDO	BLYP/STO-3G, BLYP/6-311G	Gaussian 98	Assessment of structural stability and electronic properties of substitutional Si-doped C_60_	[42]
C_59_Si	GO, ESPA, IR	PM3	-	MOPAC 2002 via CAChe 1.33	Evaluation of structural, electronic and vibrational properties of Si-doped C_60_ in comparison to pristine C_60_ C_59_Al and C_59_Ge using semiempirical method	[57]
C_48_Si_12_	GO, ESPA, CPMD	-	BLYP	CPMD code	Investigation of structural stability and electronic properties of C_48_Si_12_ heterofullerene isomers with compact Si patterns	[87]
C_30_Si_30_	GO, ESPA, CPMD	-	BLYP	CPMD code	Investigation of structural and electronic effects of positive and negative charging on highly Si-doped fullerene	[79]
C_60−n_Si_n_ (n = 1–12)	MCS, GO, ESPA	-	PW91	VASP code, DFTB+ code	Investigation of ground-state structures and stability of silicon-multisubstituted C_60_ fullerenes	[73]
C_48_Si_12_, C_36_Si_24_	GO, ESPA	-	PBE/DZP (TM)	SIESTA code	Investigation of energetic, geometrical, and electronic properties of silicon–carbon fullerene derivatives C_48_Si_12_ and C_36_Si_24_	[71]

GO—Geometry Optimization, ESPA—Electronic Structure and Properties Analysis, QR-TDDFT—Quadratic Response Time-Dependent Density Functional Theory, LR-TDDFT—Linear Response Time-Dependent Density Functional Theory, PCM—Polarizable Continuum Model, NICS—Nucleus-Independent Chemical Shift, GIAO—Gauge-Including Atomic Orbital, SCRF—Self-Consistent Reaction Field, MCS—Monte Carlo Simulation, FPDFT—First Principles Density Functional Theory, GA—Genetic Algorithm, CBG—Cage-Building Geometry, CMCS—Conformational Monte Carlo Sampling, MNDO—Modified Neglect of Differential Overlap, FPMD—First-Principles Molecular Dynamics, PF—Pair Distribution Function, CPMD—Car–Parrinello Molecular Dynamics.

**Table 5:** Selected articles on application of theoretical calculations on endohedral complexes of substitutional Si-doped C_60_ fullerenes.

Endohedral Complexes of Substitutional Si-Doped C_60_ Fullerenes							
Si-Doped Fullerene Derivative (Host)	Guest	Type of Calculation	Calculation Method		Computational Software	Aim of Study	(Ref)
			Semiempirical	DFT (Functional and Basis Set)			
C_59_Si	mH (m = 1)	GO, ESPA, E_ads_ (with BSSE)	-	PBE/LCPAO (TM)	OpenMX code	Investigation of the structural and electronic effects of endohedral atomic H encapsulation in Si-substituted C_60_ (in comparison to C_59_P, C_59_B, C_59_S, C_59_O and pristine C_60_)	[122]
C_59_Si	mH_2_ (m = 1, 2, 3, 4)	GO, ESPA, E_ads_	-	PBE/DZP (TM-NCPP)	SIESTA code	Estimation of the stability and storage capacity of molecular H_2_ in Si-substituted C_60_ via encapsulation of 1–4 H_2_ molecules (in comparison to C_59_N, C_59_B and pristine C_60_)	[58]

LCPAO—Linear Combination of Pseudo-Atomic Orbitals, BSSE—Basis Set Superposition Error Correction, GO—Geometry Optimization, ESPA—Electronic Structure and Properties Analysis.
